# Engineering stress as a motivation for filamentous virus morphology

**DOI:** 10.1016/j.bpr.2024.100181

**Published:** 2024-09-10

**Authors:** Andrew McMahon, Swetha Vijayakrishnan, Hafez El Sayyed, Danielle Groves, Michaela J. Conley, Edward Hutchinson, Nicole C. Robb

**Affiliations:** 1 Biological Physics Research Group, Clarendon Laboratory, Department of Physics, University of Oxford, Oxford, United Kingdom; 2 Kavli Institute for Nanoscience Discovery, Dorothy Crowfoot Hodgkin Building, University of Oxford, Oxford, United Kingdom; 3 Warwick Medical School, University of Warwick, Coventry, United Kingdom; 4 MRC-University of Glasgow Centre for Virus Research, University of Glasgow, Glasgow, United Kingdom

## Abstract

Many viruses are pleomorphic in shape and size, with pleomorphism often thought to correlate with infectivity, pathogenicity, or virus survival. For example, influenza and respiratory syncytial virus particles range in size from small spherical virions to filaments reaching many micrometers in length. We have used a pressure vessel model to investigate how the length and width of spherical and filamentous virions can vary for a given critical stress and fluorescence super-resolution microscopy along with image analysis tools to fit imaged influenza viruses to the model. We have shown that influenza virion dimensions fit within the theoretical limits of the model, suggesting that filament formation may be a way to increase an individual virus’s volume without particle rupture. We have also used cryoelectron microscopy to investigate influenza and respiratory syncytial virus dimensions at the extrema of the model and used the pressure vessel model to explain the lack of alternative virus particle geometries. Our approach offers insight into the possible purpose of filamentous virus morphology and is applicable to a wide range of other biological entities, including bacteria and fungi.

## Why it matters

Many viruses form particles that vary in size and shape, including small spheres or long filamentous structures that can reach many microns in length. While virion structure is of interest not only in the context of virus assembly but also because pleomorphic variations may correlate with infectivity and pathogenicity, filament formation is not well understood, and viral filaments are relatively understudied. In this paper, we used a pressure vessel model and microscopy to investigate the relationship between virion dimensions and engineering stress and concluded that viral filaments offer an optimal shape that allows viruses to have a larger volume than they could in any alternative geometry, thus offering new insight into viral filament formation.

## Introduction

A pressure vessel is designed to hold liquids or gases at substantially higher or lower pressures than ambient pressure. A high pressure difference requires the correct design in order to avoid catastrophic failures, and pressure vessel models (1) are used to assess engineering stress development and whether vessels of a known geometry will be able to sustain the applied internal pressure. Stress is the force applied to a material divided by the area over which the force acts before deformation occurs. By equating the force due to pressure and the equal and opposite force from the stress, a relation between stress, pressure, and the dimensions of a given cross section of the container can be derived. In chemical engineering, pressure vessel models using theoretical reaction pressures and the critical stress of a material are used to determine the geometries of reaction vessels (size and wall thickness) so that their operation falls within allowed safety margins. We set out to investigate whether pressure vessel models could be applied to filamentous biological structures and provide insight into pressure-stress relations, using size data from images of viruses and bacteria to test our model.

A filamentous, or elongated, morphology is a common feature of many biological microorganisms and can be observed in multiple species of fungi, bacteria, and viruses. Viruses in particular can be extremely pleomorphic; in the case of influenza, particles can range from small spheres in the region of 100 nm, to extended bacilliform particles, to long filaments, which reach tens of microns in length (2,3). The role of filaments in influenza is poorly understood, with current suggestions for why a filamentous morphology occurs including the increased ability of filaments to penetrate mucus layers (4); their increased directionality of travel on mucus layers compared to spherical forms, which enables filaments to infect cells further from the original host cell in a shorter time (5); their increased resilience to UV radiation (6); or their increased ability to evade antibodies due to their increased number of surface proteins per virion (7). In contrast, the larger surface area of a filament in comparison to a spherical particle means that significantly more proteins are required to form each progeny virion, and so overall, far fewer filamentous virions than spherical can be produced by each infected cell, which may explain why spherical virions are also formed during new virion production. In the case of respiratory syncytial virus (RSV), another pathogenic virus that has been observed to have a filamentous form, there is evidence that filamentous virions represent the infectious form of the virus required for fusion of the virus with the host cell (8).

In this study, we modeled filaments as cylindrical particles with hemispherical caps and used known results from thin-walled pressure vessels (1) to form a theory of how the major axis of a filament would change in relation to its minor axis given a critical stress (9). This derived relationship was tested using microscopy measurements of the length and width of influenza and RSV. We found that a pressure vessel model of a filament gave a quadratic relation between the major and minor axes that fitted well to the size measurements taken for both viruses, suggesting that filament formation may be a way of increasing the volume of a virus without leading to particle rupture. Overall, our modeling provides insights into how pressure and stress may influence the size and shape of viruses and other biological organisms.

## Materials and methods

### Calculating a theoretical major/minor axis relation

A thin wall pressure model was used to derive the three normal stresses i.e., stresses formed when the direction of the deforming force is perpendicular to the cross-sectional area of the body. The force due to pressure from the inside of a filament was equated with the force due to the stress in the walls of the filament due to Newton’s third law, with the filament modeled as a cylinder with hemispherical caps. This gave principle stress/pressure relations for the longitudinal stress, σ_1_, hoop stress, σ_2_, and normal/external stress, σ_3_:

(1) $$\begin{array}{c} \sigma_{1}=\frac{P}{t}\frac{\left(\pi r^{2}+2\text{Lr}\right)}{\left(2\pi r+2L\right)} \end{array}\text{,}$$

(2) $$\begin{array}{c} \sigma_{2}=P\frac{r}{2t},and \end{array}$$

(3) $$\begin{array}{c} \sigma_{3}=-P\text{,} \end{array}$$

where P is the difference in pressure over the surface (P_internal_ – P_external_), t is the thickness of the lipid layer, r is the radius of the cylinder and hemispheres, and L is the length of the cylinder. In the limit L→0, Eq. 1 tends to the normal hoop stress of a sphere, and, as L→∞, it tends to the hoop stress of a cylindrical pipe.

Assuming the breaking stress occurs when σ_1_ = σ_max_, σ_2_ = σ_max_, or σ_3_ = σ_max_ and using r = r_∞_ when L→ꝏ, this gives us

(4) $$\begin{array}{c} r_{\infty }=\frac{\sigma_{\max}t}{P}\text{,} \end{array}$$

as σ_1_ is always the greatest of the stress components.

This gives the relationship between L (the length of the cylindrical segment) and r, the radius, in terms of the radius of an infinitely long filament, $r_{\infty }$, of

(5) $$\begin{array}{c} L=\frac{2\pi rr_{\infty }-\;\pi r^{2}}{2\left(r-r_{\infty }\right)}\text{,} \end{array}$$

which, using the major axis length, m_1_ = L + 2r, and the minor axis length, m_2_ = 2r = m_1_ – L, gives us the relation to compare experimentally between the major and minor axes:

(6) $$\begin{array}{c} m_{1}=m_{2}+\frac{\pi m_{2}\left(r_{\infty }-\;\frac{{m_{2}}^{2}}{4}\right)}{m_{2}-2r_{\infty }} \end{array}$$

Within the model, several assumptions were made.

1) The internal pressure was uniform across different virus particles of the same species.
2) The surface environment across the surface of virus particles was similar and the material was isotropic, and thus the maximum stress was constant.
3) The bending stress in the virus membrane was not considered in the thin wall approximation.
4) When cutting shapes to find a cross section, perpendicular lines on the neutral axis remain perpendicular after cutting.

### Deriving a relation between spherical and filamentous virions with a non-negligible membrane thickness

Assuming the critical shear stress of the material is constant across filaments and spheres, we equated the maximum shear stress from Lamé’s equations (10) for a sphere and a cylinder:

(7) $$\begin{array}{c} \frac{3p_{o}{b_{\text{sphere}}}^{3}-p_{i}\left(2{a_{\text{sphere}}}^{3}+{b_{\text{sphere}}}^{3}\right)}{2\left({a_{\text{sphere}}}^{3}-{b_{\text{sphere}}}^{3}\right)}=\frac{p_{i}\left(a^{2}+b^{2}\right)-{2p}_{o}b^{2}}{b^{2}-a^{2}}\text{,} \end{array}$$

where b_sphere_ is the outer radius of spherical particles, a_sphere_ is the inner radius of spherical particles, b is the outer radius of filamentous particles, a is the inner radius of filamentous particles, p_o_ is the external particle pressure, and p_i_ is the internal particle pressure.

From this, we derived a relation between filament and spherical virion sizes that accounts for bending stresses given that the internal pressure is greater than the external pressure:

(8) $$\begin{array}{c} \frac{\left(b_{\text{sphere}}^{3}+2a_{\text{sphere}}^{3}\right)\left(b^{2}-a^{2}\right)}{2\left(b_{\text{sphere}}^{3}-a_{\text{sphere}}^{3}\right)\left(b^{2}+a^{2}\right)}=1 \end{array}\text{.}$$

## Results

### The derivation of a relation between the major and minor axes of filaments

**Fig. 1:**
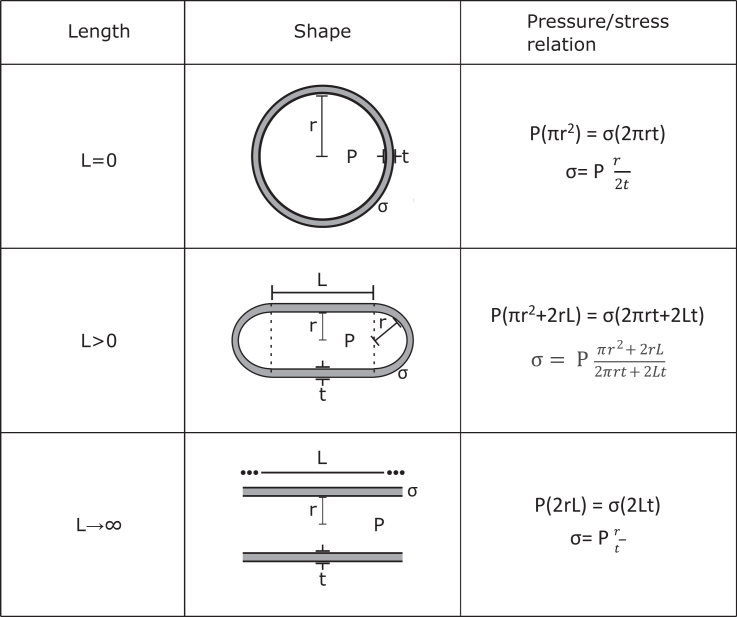
Schematics of the cross sections of interest and the pressure/stress relations derived for increasing lengths of virus particles. Viruses are modeled as cylinders with hemispherical caps. For different lengths of cylinders, L = 0, L > 0, and L→ꝏ, the pressure/stress relation is given along with the cross section of interest. In the limits of L→0 and L→ꝏ, the model used for filamentous viruses tends to the known results for spheres and pipes of an infinite length.

In order to investigate any physical motivation for filamentous morphologies, a pressure vessel model was formed. We did this by modeling the virus as a cylinder of length L and radius r with hemispherical caps of radius, r (Fig. 1). Taking a cross section of this and equating the force due to pressure with the oppositely directed force due to the stress in the walls of the filament, we could derive the known relation for a pressure vessel (materials and methods, Eq. 1). This pressure stress relation was valid for viruses of any size, from a sphere to a filament. The relation showed that the stress increased as the radius increased for spherical morphologies. However, for a filamentous morphology, the stress in the membrane did not continue to increase as the length increased. Instead, as the filament length tends to an infinite length, the stress tends to a value twice that of the spherical particle with the same radius (Fig. 1).

Once our pressure stress relation was formed, an experimentally testable relation was required. With the assumptions that there was a maximal allowed stress (as membranes rupture at high stress) and a constant internal pressure (as different pleiomorphic viruses bud from the same cells)m we derived a relation between the minor axis, m_2,_ and the major axis, m_1_, of viral particles (materials and methods, Eq. 6):

(9) $$\begin{array}{c} m_{1}=m_{2}+\frac{\pi m_{2}\left(r_{\infty }-\;\frac{{m_{2}}^{2}}{4}\right)}{m_{2}-2r_{\infty }}\text{.} \end{array}$$

This relation shows a quadratic relation between the major and minor axes for a given value of r_∞_ and that as the major axis increases, the minor axis decreases. As m_1_ tends to an infinite length, m_2_ tends to half the value when m_2_ = m_1_. This provides us with a tractable relation that can be applied to the study of filamentous virus morphology.

### The application of the pressure vessel model to biological images

**Fig. 2:**
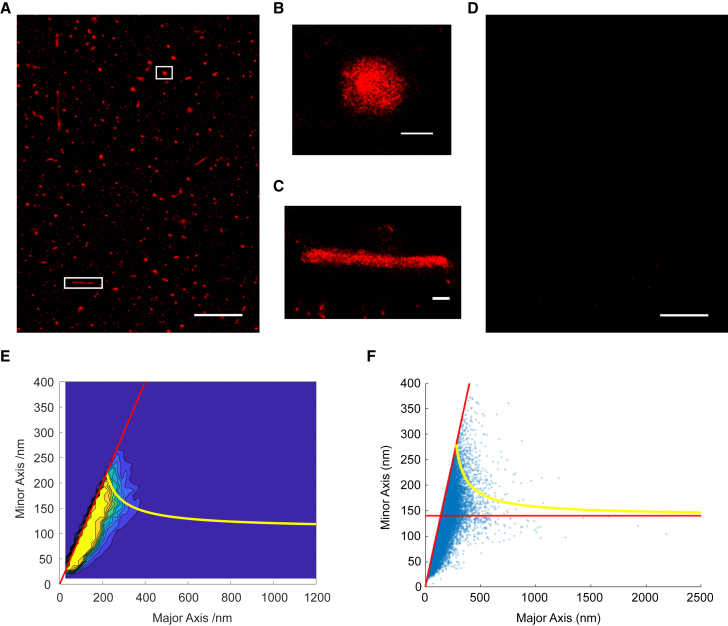
Super-resolution imaging of spherical and filamentous influenza particles fit against the predicted theory from pressure vessel analysis. (*A*) A representative direct stochastic optical reconstruction microscopy (dSTORM) field of view (FOV) of labeled A/Udorn/72 influenza where the hemagglutinin protein is imaged in the red channel. Scale bar, 10 *μ*m. (*B* and *C*) Zoomed-in images from (*A*) showing individual filaments and spherical particles. Scale bar, 200 nm. (*D*) A representative dSTORM FOV of a virus-negative sample, imaged in the red channel. Scale bar, 10 *μ*m. (*E*) The major/minor axis contour plot with lines at major axis = minor axis (*red*) and with the line as given in Eq. 6 describing the derived allowable relation between major and minor axes with r_0_ = 70 nm (*yellow*) with a maximum frequency of 100 for clarity. (*F*) The major/minor axis scatterplot with lines at minor axis = 140 nm (*red*) and major axis = minor axis (*red*) and with the line as given in Eq. 6 describing the derived allowable relation between major and minor axes with r_0_ = 70 nm (*yellow*).

To experimentally test the relation between major and minor axis lengths of filaments, we performed super-resolution imaging of the influenza virus A/Udorn/72, a strain with a well-characterized spherical and filamentous phenotype (11). Virus particles were immobilized, fixed, and immunolabeled, and direct stochastic optical reconstruction microscopy (dSTORM) was carried out to obtain high-resolution reconstructed images, with a localization precision of 7.3 nm (Fig. 2 *A*) (11). Prior to this work, we carried out several control experiments, confirming that our methodology of drying and immunolabeling virus samples does not adversely influence the resulting images (11). Negative-stain electron microscopy images of our virus stocks also confirmed that virus particles were intact and did not appear to be aggregated (Fig. S1), giving us confidence that the majority of particles that we imaged were intact, non-aggregated virions. Both spherical (Fig. 2 *B*) and filamentous (Fig. 2 *C*) particles greater than 250 nm in length were observed in the resulting images. Minimal signal was observed in a negative control consisting of cell culture media lacking virus particles, confirming that the virus particles were specifically labeled (Fig. 2 *D*).

Measurements of the major and minor axes of particles were then taken from the super-resolution images by clustering the localizations using the DBScan algorithm and then fitting a confidence ellipse to the clustered data. A total of 41,754 viruses were measured from 46 fields of view (FOVs). The final bivariate histogram (Fig. 2 *E*) was produced by subtracting the negative FOV distributions (Fig. S2) from the positive FOV distributions. The bivariate histogram showed that the largest virions primarily appeared in two directions—along the line with the major axis equal to the minor axis and the line with the minor axis equal to 140 nm (Fig. 2 *E*). Additionally, a scatterplot of the major axis against the minor axis, with the lines m_1_ = m_2_ and m_2_ = 2r_∞_ highlighted in red, and the derived relation between the minor axis and major axis (Eq. 6), with r_∞_ = 70 nm plotted in yellow, showed the boundary of the points following the derived relation (Fig. 2 *F*).

To compare our findings on viruses to another biological system, we also imaged *E. coli* bacteria. *E. coli* is a straight, rod-shaped bacterium approximately 1–3 *μ*m in length. In a natural, unstressed population, approximately 10% of the cells form filamentous structures (12); however, when they are stressed, the proportion of filamentous structures increases, which suggests that filaments may play a role in protecting the bacteria from predation, phagocytosis, and other stressors (12,13,14). Here, we imaged *E. coli* (Fig. S3 *A*) and segmented the individual bacteria within the images to measure their lengths and widths (Fig. S3 *B*). A bivariate histogram of these data showed a decrease in the width of the cells as their lengths increased, and a scatterplot with the modeled maximum size (*yellow*) with an r_0_ of 1.1 *μ*m (*red*) shows the main density of cells fitting the model with very good agreement (Fig. S3, *C*–*E*). Taken together, our results from images of both filamentous influenza virus and *E. coli* suggest that elongation of viruses and bacteria is accompanied by a narrowing of the particles along the minor axis, as predicted by our model.

### Filamentous virus particles tend to half the width of spherical virions

**Fig. 3:**
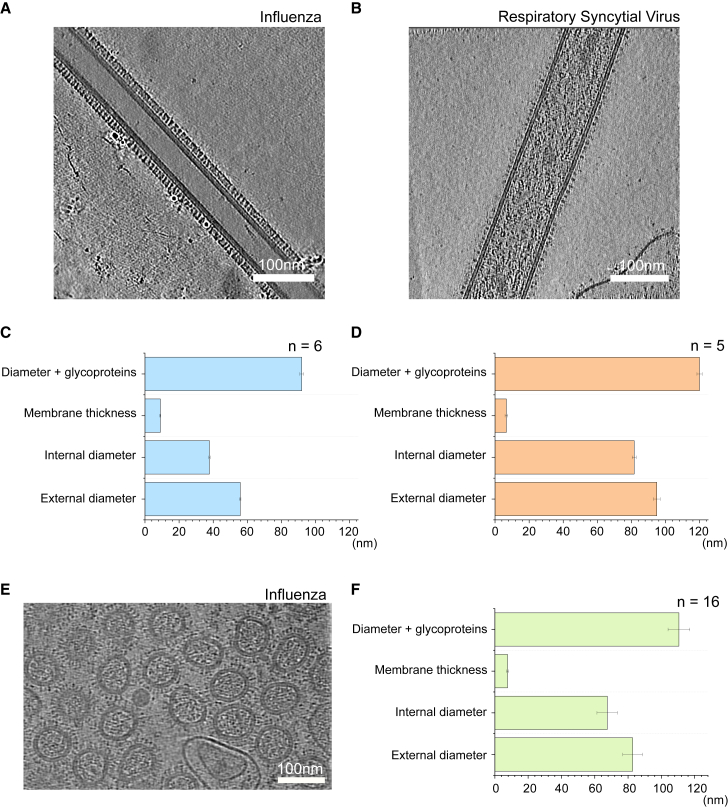
Cryo-electron tomography of filamentous and spherical virus particles and measurements of their diameter and membrane thickness. (*A*) Representative tomogram of filamentous A/Udorn/72 influenza virus. Scale bar, 100 nm. (*B*) Representative tomogram of filamentous A2 respiratory syncytial virus. Scale bar, 100 nm. (*C* and *D*) Bar graphs of the average outer and inner diameters and the membrane thicknesses of the two filamentous viruses. (*E*) Representative tomogram of spherical A/WSN/33 influenza virus particles. Scale bar, 100 nm. (*F*) Bar graphs of the average outer and inner diameters and the membrane thicknesses of A/WSN/33 spheres.

To further investigate the application of our derived pressure stress model to filamentous viruses, we looked at the extrema of the model when the filaments were very long, i.e., microns in length (and the pressure stress relation can be modeled as that of a pipe), against which we could compare literature values for spherical virions. Our model predicted that the radius of filaments should be half the radius of the largest allowed spherical particles. In order to get experimental data to support our model, purified preparations of the A/Udorn/72 influenza virus and the A2 strain of RSV were imaged using cryoelectron tomography (cryo-ET) to produce tomograms of filaments with nanometer resolution (Fig. 3, *A* and *B*). By measuring the widths of individual filaments in the resulting images, we found the average external diameter (with and without surface glycoproteins), internal diameter, and thickness of the membrane for six influenza filaments and five RSV filaments.

This analysis provided measurements of 91.9 ± 1.1, 9.0 ± 0.6, 37.8 ± 0.4, and 55.8 ± 0.5 nm for the external diameter including glycoproteins, membrane thickness, internal diameter of the influenza filament membrane, and external diameter without glycoproteins, respectively (Fig. 3 *C*). This is in broad agreement with previously published values for the width of influenza filaments, for example, a study that measured 74.7 nm to the tips of the glycoproteins (15). Images of RSV filaments revealed that they were wider than influenza filaments, with average measurements for the external diameter including glycoproteins, membrane thickness, external diameter without glycoproteins, and internal diameter of 119.9 ± 1.5, 6.7 ± 0.8, 81.6 ± 1.2, and 94.9 ± 1.0 nm, respectively (Fig. 3 *D*). Similarly to the influenza measurements, these figures are in agreement with previously published values of between 71 and 166 nm for the width of RSV filaments (16).

Next, we compared our measured values from viral filaments with similar measurements for purified spherical influenza virions. Cryo-ET analysis of spherical A/WSN/33 influenza virions provided measurements of 110.6 ± 6.6, 7.6 ± 0.6, 67.5 ± 6.2, and 82.7 ± 5.9 nm for the external diameter including glycoproteins, membrane thickness, internal diameter of the membrane, and external diameter without glycoproteins, respectively (*n* = 16) (Fig. 3, *E* and *F*). We were unable to obtain cryo-ET images of purified RSV spheres because the purification procedure failed to produce intact spherical particles; however, negative-stain electron microscopy of unpurified RSV virions (Fig. S4) provided images of RSV particles with average diameters of 356 ± 130 nm (*n* = 20). Both sets of our measurements are in good agreement with previously published values for the diameter of spherical virions (84–170 nm for influenza (17) and 150–500 nm for RSV (18)). Comparison of our measured values for filaments with our values for spherical particle diameter provides good agreement with our model that predicts the filament membrane diameter to be approximately half the size of the maximum spherical membrane diameter. We also found that the membrane thickness of RSV was 7.6% of its diameter at its neutral axis (taken as the center of the viral membrane); for influenza, it was 19.6%. This puts RSV filaments comfortably within the thin wall approximation and influenza at the extreme of the model.

Although we have not included the effect of bending stress in our model, the measurements obtained from these cryo-ET images allow us to compare experimental and theoretical values for filaments and spheres taking bending stress into account. First, we used Lamé’s equations to derive a relation between filament and spherical virion sizes that accounts for bending stresses for a constant critical shear stress, assuming that the internal pressure of virions is greater than the external pressure (see materials and methods). Using this relation and inputting the data from the electron tomograms of influenza (Fig. 3 *C* and *F*), we computed a value of 0.84 ± 0.08, which is within two standard deviations of the expected value of one, suggesting that the dimensions of influenza virions fit the pressure vessel model well when taking bending stresses into consideration.

## Discussion

In this work, we have presented a pressure vessel model to explain the physical basis of filamentous virus morphology. A model was formed that related the stress, internal pressure, and length scales of a virus particle. It showed that the stress increased with the radius of a spherical virion and that a filament is a method of avoiding the size constraint that this imposes upon virion size due to the bursting stress of the membrane. We used microscopy images of influenza viruses to test our proposed model and confirmed that influenza virions tended to a decreased minor axis at large major axis length scales. Our model’s prediction that filamentous virions should have half the diameter of spherical virions also appears to fit experimental data for both influenza and a second filamentous virus, RSV. We were also able to provide similar data from images of the bacteria *E. coli*, which suggests that the model is applicable to other cells and biological entities. This may explain why some viral and bacterial species can grow up to tens of microns in length without rupture due to their internal pressure becoming too large.

**Fig. 4:**
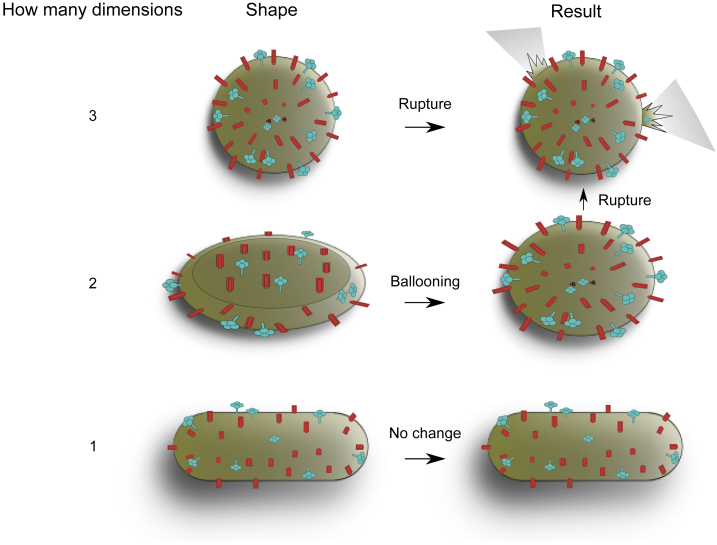
Schematic of the proposed shapes formed from symmetric enlargement in three, two, or one dimensions and the outcome for virions with those geometries. The three possible shapes of the virus when they are enlarged symmetrically in either one, two, or three dimensions are shown. Three-dimensional enlargement is predicted to result in virion rupture when the maximum stress is reached, while growth in two dimensions is predicted to result in ballooning that will lead to a similar fate. Our data suggest that a filamentous shape with enlargement along a single plane is not likely to encounter this issue.

Our derived model allows us to consider the potential different geometries that could be formed by virus particles. As smooth, symmetrical geometries minimize stress concentrations, we have considered the different geometries that could be formed as particles emerge symmetrically from the membrane in one, two, or three dimensions (1D, 2D, or 3D, respectively). In 1D, a filament is formed, in 2D a circular sheet, and in 3D a sphere (Fig. 4). We have excluded the possibility of nonuniform geometries, as these would produce local maxima in the stress of the membrane due to their local cross section and so are likely not formed due to the limitation of the rupture stress of the viral membrane. Our pressure model suggests that the increasing stress as spherical virus particles are enlarged in 3D to an infinite size would lead to rupture of the membrane, thus explaining the absence of large spherical viruses (Fig. 4, *top row*). Li et al. showed the lipid bilayer to be an easily deformable, nonrigid container (19), which suggests that 2D enlargement would also not be stable without further support to the membrane and that particles would “balloon” to a 3D spherical geometry upon budding from cells, also leading to rupture (Fig. 4, *middle row*). A filamentous structure, with elongation along just a single plane, will not encounter this problem (Fig. 4, *bottom row*). This model may therefore explain why virus particles exist as either small spheres or long filaments and have not been observed to bud in disk shapes along two principle directions.

Although our model predicts the allowed dimensions of the virus particles, it does not encompass the effects that the pool of host membrane at budding sites might have on these dimensions. This interaction may explain the ability of RSV viruses to form branched virions. Each individual branch in RSV has a very similar geometry to a viral filament, with the exception of the branching points, and so we believe that these are largely covered by our model. At the branch point, however, the geometry still appears to be relatively smooth (8), and so while these points are likely to have slightly higher stresses, we assume that the forces involved do not cause shear. The recently described “giant viruses,” such as those belonging to the family *Mimiviridae*, which are distinguished by their large capsid diameters (200–400 nm), could potentially pose an exception to our model; however, we note some points of potential importance, namely that giant viruses are contained within rigid protein capsids and that exit from the host cell occurs via lysis rather than budding.

Our model has a number of limitations, including the following: the pressure throughout filaments and particles may not be fully uniform; structural proteins within the lipid membrane may change the maximum stress of filaments (assumed to be a constant); the walls have a finite thickness, and so the assumption that the radius of the lipid layer is equal to the radius of the inside of the virus is not perfect; bending stress is not included in the model (see below); and the model cannot explain the mechanism of filament formation or why some strains of viruses form filaments but others do not. In spite of these caveats, our model suggests filament formation to be a method through which enveloped viruses can become larger than the limit placed upon them from the stress of a lipid bilayer. As larger viruses require more material from the cell to assemble, and thus their formation decreases the total number of virions produced, this suggests that filament formation must offer the virus an advantage. Several studies have suggested that this is indeed the case, with filamentous viruses having an advantage in transmission (e.g., through containing multiple copies of the genome) (6) by increasing the virus particle surface area and thus making filaments less susceptible to neutralizing antibodies (7), increasing the ability of filaments to penetrate mucus layers (4), or increasing the resilience to UV radiation (6).

As mentioned above, the full complexity of the viral system is not completely captured by our model and images. Prior to rupture, the membrane of the virus is expected to detach from the underlying protein matrix layer. We would expect that the loss of the matrix layer will result in a decrease in the critical shear stress for the virion, which in turn would increase the probability of rupture. We have also not included bending stress in our model, which would be expected to result in a higher stress in particles with a greater curvature than predicted in our calculations, which would in turn lead to a smaller diameter of filamentous virions. In order to investigate this further, we derived a relation between the dimensions of spherical and filamentous virions taking bending stress into consideration and compared the theoretical values obtained to our experimental cryo-ET measurements of influenza virions. The small difference between theoretical and experimental values suggests that the dimensions of influenza virions do fit the pressure vessel model well when taking bending stresses into consideration. Due to the relatively low-throughput nature of the cryo-ET method, we have only carried out these measurements on a relatively small number of virus particles and may, therefore, have not captured the full variability of these viruses; however, we are confident that the inclusion of further images will not alter our main conclusion: that engineering stress is a key motivation for filamentous virus morphology.

In conclusion, modeling of viruses as a thin-walled pressure vessel with a maximum stress leads to the conclusion that filaments are formed as a method of increasing the size of virions while staying below this maximum stress. The model can be used to explain why virus particles exist as either small spheres or long filaments rather than in nonuniform geometries or disk-shaped particles. Furthermore, the model is broadly applicable to a wide range of biological organisms that form elongated filamentous structures.

## Acknowledgments

This research was supported by Royal Society Dorothy Hodgkin Research Fellowship DKR00620 and Research Grant for Research Fellows RGF∖R1∖180054 (to N.C.R.), 10.13039/501100000265Medical Research Council core grant MC_UU_12014/7 (to S.V.), UK Biotechnology and Biological Sciences Research Council grant BB/X015637/1 (to H.E.S.), a Medical Research Council-Doctoral Training Partnership-funded studentship (to D.G.), and a Medical Research Council Career Development Award MR/N008618/1 (to E.H.). We would like to thank Dr. James Streetly and Prof. David Bhella for assistance with cryo-tomography and Dr. Saskia Bakker for assistance with the negative-stain electron microscopy.

## Author contributions

A.M. conceived the project, acquired data, performed the analysis, and drafted the manuscript; H.E.S. contributed to image acquisition; S.V., M.J.C., and D.G. acquired data and contributed to image acquisition; E.H. contributed to funding acquisition and supervision; and N.C.R. contributed to funding acquisition and supervision, drafted figures and revised the manuscript.

## Declaration of interests

The authors declare no competing interests.

## Supporting material

Document S1. Figures S1–S4 and supporting materials and methods

Document S2. Article plus supporting material
