# Supplementary material for: Engineering stress as a motivation for filamentous virus morphology
Source: Biophys Rep (N Y). 2024 Sep 10;4(4):100181. doi: 10.1016/j.bpr.2024.100181 (PMC11447354; doi:10.1016/j.bpr.2024.100181)
Supplement: Document S1. Figures S1–S4 and supporting materials and methods [file mmc1.pdf]

Biophysical Reports, Volume *4*

Supplemental information

Engineering stress as a motivation for filamentous virus morphology

Andrew McMahon, Swetha Vijayakrishnan, Hafez El Sayyed, Danielle Groves, Michaela J. Conley, Edward Hutchinson, and Nicole C. Robb

Supplementary Material

Engineering stress as a motivation for filamentous virus morphology

Andrew McMahon[1,2,3,*], Swetha Vijayakrishnan[4], Hafez El Sayyed[1,2], Danielle Groves[3], Michaela J. Conley[4], Edward Hutchinson[4], and Nicole C. Robb[1,3,*]

[1]Biological Physics Research Group, Clarendon Laboratory, Department of Physics, University of Oxford, Oxford, OX1 3PU, United Kingdom

[2]Kavli Institute for Nanoscience Discovery, Dorothy Crowfoot Hodgkin Building, University of Oxford, South Parks Rd, Oxford, OX1 3QU, United Kingdom

[3]Warwick Medical School, University of Warwick, Coventry, CV4 7AL, United Kingdom

[4]MRC-University of Glasgow Centre for Virus Research, University of Glasgow, Glasgow, G61 1QH, United Kingdom

*To whom correspondence should be addressed: Nicole.Robb@warwick.ac.uk and Andrew.McMahon@warwick.ac.uk

Supplementary Methods

Viruses. The influenza strain A/Udorn/72 (H3N2) (Udorn) was a kind gift from Professor Ervin Fodor, University of Oxford and was grown in Madin-Darby canine kidney (MDCK) cells as previously described [1]. Udorn virions have been shown to exhibit both a filamentous and spherical morphology [1]. Respiratory Syncytial Virus strain A2 (RSV) (ATCC, VR-1540) was grown in HEp-2 cells as previously described [2]. RSV virions have also been shown to exhibit both a filamentous and spherical morphology [3]. Spherical influenza A/WSN/33 (H1N1) (WSN) was prepared by reverse genetics, as previously described [4], and propagated on MDCK cells.

Super-resolution imaging and analysis. A/Udorn/72 influenza virus particles within cell supernatant (or cell media only as a negative control) were imaged using direct stochastic optical reconstruction microsopy (dSTORM) and analysed as described previously [1]. Briefly, viruses were dried onto a poly-L-lysine treated glass coverslip, fixed with 4% formaldehyde (Thermo Scientific), permeabilized with 0.5% Triton-X-100 (MP Biomedicals) and immunolabelled. We previously found that including a permeabilization step resulted in a higher number of labelled particles in our images, regardless of whether the target protein was internal or external [1]. A primary antibody against the haemagglutinin protein (Hc83x, a kind gift from Stephen Wharton, Francis Crick Institute) and a secondary antibody labelled with Alexa647 (Invitrogen) were used. The viruses were imaged on a commercially available Nanoimager fluorescence microscope (Oxford Nanoimaging) using total internal reflection fluorescence (TIRF) microscopy. The laser illumination was focused at an angle of 53° with respect to the default position. Images of a field of view (FOV) measuring 80 x 50 µm were taken with an exposure time of 30 ms at a laser intensity of up to 780 kW/cm^2, and movies of between 5,000 and 15,000 frames were taken.

The signals in each FOV were detected and fit to a 2D Gaussian in each frame using the analysis software from Oxford Nanoimaging, drift corrected via phase correlation between frames, and exported. Each FOV was clustered using the sklearn clustering library implementation of the DBScan clustering algorithm with an epsilon of 30 nm and a minimum cluster size of 200 nm [1]. Using the confidence_ellipse library, a confidence ellipse was fit with the standard deviation of the ellipse set as 2.0. For the width of particles, the scipy.spatial implementation of the convex hull method was used. The major and minor axes of the ellipse were taken as the size of the viral particles.

Cryo-electron tomograhy imaging and analysis. Preparation of grids for cryo-electron tomography (cryo-ET) and imaging was carried out as described previously [5]. Briefly, Madin-Darby canine kidney (MDCK) and adenocarcinomic human alveolar epithelial (A549) cells were directly seeded on cryo-EM grids prior to infection with human RSV (strain A2-RSV) or A/Udorn/72 (H3N2) at an MOI of 1 and incubated for a further 24 h (Udorn) or 72 h (RSV A2). Stocks of A2-RSV and Udorn virus were produced in house as previously described [5, 6]. Stocks of WSN were prepared for cryo-electron tomography as previously described [7]. Briefly, twenty confluent T150 flasks of MDCK cells were infected with WSN at an MOI of 0.001 PFU/cell and maintained in serum-free DMEM at 37°C. After two days, the growth medium was clarified twice by low-speed centrifugation and then virions were concentrated by ultracentrifugation at 112,000 × *g* for 90 minutes at 4°C through a cushion of 10% iodixanol (supplied as OptiPrep, Sigma) in NTC (0.1 M NaCl, 20 mM Tris-HCl pH 7.4, 5 mM $CaCl_2$). The pellet was resuspended in NTC and separated through a 10 – 35% gradient of iodixanol in NTC, prepared using a Gradient Master (BioComp Instruments), by ultracentrifugation at 4 °C for 150 min at 209,000 × *g*. WSN virions, visible as a thick milky band on the gradient, were drawn off by side-puncture, pelleted by ultracentrifugation through NTC at 4 °C for 60 min at 154,000 × *g*, and resuspended in 80 µl NTC.

Grids were plunge frozen as follows. Briefly, RSV-infected and Udorn-infected grids were supplemented with 3 µl of 5 nm colloidal gold bead suspension, and purified WSN virus (4 µl) was

supplemented with 1 µl of 10 nm colloidal gold bead suspension (BBI Solutions, United Kingdom). Grids were then transferred to a Vitrobot Mk IV (Thermo Fisher Scientific), blotted for four or five seconds and immediately plunged into a bath of liquid ethane. Tilt-series imaging was performed at the UK electron bio-imaging centre at Diamond Light Source (eBIC) on a Titan Krios microscope (Thermo Fisher Scientific, Germany) equipped with a Gatan BioQuantum K2 energy filtered direct detection camera, and at the Scottish Centre for Macromolecular imaging (SCMI) on a JEOL CRYO ARM 300 (JEOL, Japan) equipped with an energy filter and a DE64 direct electron camera (Direct Electron, USA). Tilt series alignment and tomogram reconstruction was performed and visualized using the IMOD software package [8]. Reconstruction was carried out using weighted back projection followed by denoising using Topaz [9]. Figures were prepared by averaging 10 tomogram sections using IMOD's 3dmod slicer routine.

The contrast of each tomogram taken was adjusted to make the boundaries of the filaments clear. The outer and inner diameters of each filament in the acquired tomograms were measured in 5 different locations with the line tool of ImageJ. These were used to calculate the average width of each filament and the error in these measurements. From the diameter information, the wall thickness was calculated with the error in wall thickness calculated through error propagation.

Negative Stain Electron Microscopy. Carbon coated formvar grids were floated on droplets of the virus samples for 5 min followed sequentially by 5 min on a droplet of water and then on 1% uranyl acetate. The grids were blotted and allowed to dry before examination in a JEOL 2100 Plus microscope operating at 200 kV. We measured the size (average of major and minor axes) of particles using the wand tool in ImageJ.

Bacteria imaging and analysis. Images of Escherichia coli (E. coli) bacteria were acquired as described previously [10]. Briefly, bacteria were grown to their exponential phase when the OD600 was measured to be ~0.2 and imaged using brightfield microscopy. Brightfield images were illuminated using a white LED light source (CoolLED pE-100). Collected light was passed back through the multi-notch filter with transmission windows at 439±15 nm, 21±17 nm, and 605±25 nm, with an achromatic doublet lens (AC508-300-A, ThorLabs, Newton, New Jersey, USA) forming an image on an EMCCD camera (iXon 897 Ultra, Andor Technology Ltd, Belfast, UK). Image acquisition was performed using the software package Andor SOLIS (Andor Technology, Belfast, UK).

Bacterial image analysis was carried out using bacterial segmentation software. Cells were segmented using a convolutional neural network trained on manually labelled data, dividing and incomplete cells were excluded from the segmentation, and then imperfect segmentations were cleaned up manually. From the segmentation mask, the lengths and widths of cells were extracted.

Supporting References

1. McMahon, A., et al., *High-throughput super-resolution analysis of influenza virus pleomorphism reveals insights into viral spatial organization.* PLoS Pathog, 2023. **19**(6): p. e1011484.
2. Haney, J., et al., *Coinfection by influenza A virus and respiratory syncytial virus produces hybrid virus particles.* Nat Microbiol, 2022. **7**(11): p. 1879-1890.
3. Ke, Z., et al., *The Morphology and Assembly of Respiratory Syncytial Virus Revealed by Cryo-Electron Tomography.* Viruses, 2018. **10**(8).
4. Fodor, E., et al., *Rescue of influenza A virus from recombinant DNA.* J Virol, 1999. **73**(11): p. 9679-82.

5. Vijayakrishnan, S., et al., *Cryotomography of budding influenza A virus reveals filaments with diverse morphologies that mostly do not bear a genome at their distal end.* PLoS Pathog, 2013. **9**(6): p. e1003413.
6. Conley, M.J., et al., *Helical ordering of envelope-associated proteins and glycoproteins in respiratory syncytial virus.* EMBO J, 2022. **41**(3): p. e109728.
7. Hutchinson, E.C. and M. Stegmann, *Purification and Proteomics of Influenza Virions.* Methods Mol Biol, 2018. **1836**: p. 89-120.
8. Kremer, J.R., D.N. Mastronarde, and J.R. McIntosh, *Computer visualization of three-dimensional image data using IMOD.* J Struct Biol, 1996. **116**(1): p. 71-6.
9. Bepler, T., et al., *Topaz-Denoise: general deep denoising models for cryoEM and cryoET.* Nat Commun, 2020. **11**(1): p. 5208.
10. El Sayyed, H., et al., *Single-molecule tracking reveals the functional allocation, in vivo interactions, and spatial organization of universal transcription factor NusG.* Mol Cell, 2024. **84**(5): p. 926-937 e4.

Supplementary Figures

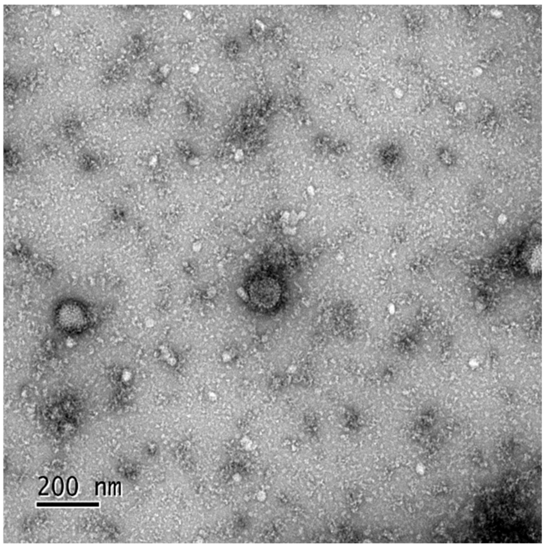

Sup. Fig. 1: Negative stain electron microscopy (EM) image of virus particles showing them to be intact and not aggregated. Negative stain EM image of A/Udorn/72 virions. Scale bar 200 nm.

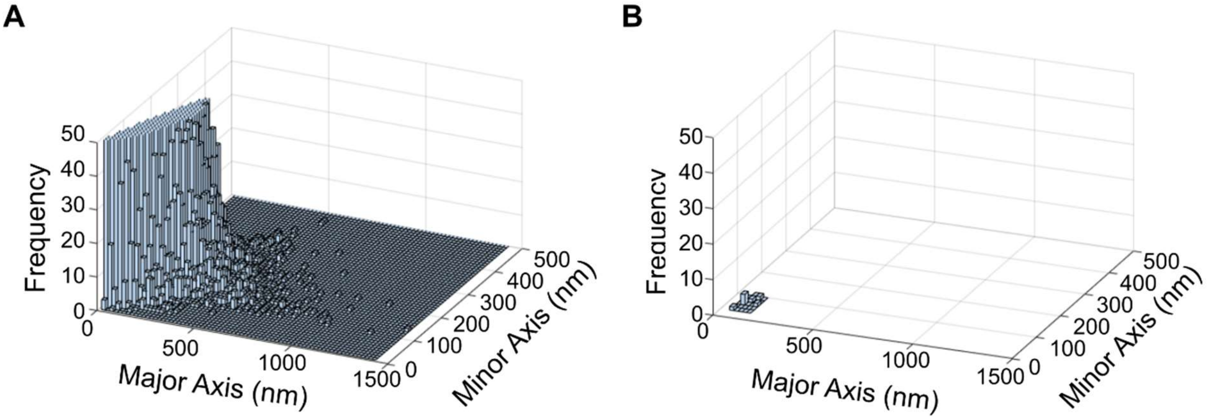

Sup. Fig. 2: Super-resolution imaging of spherical and filamentous influenza particles fit against the predicted theory from pressure vessel analysis. A) A bivariate histogram of the major/minor axis lengths. The frequency decreases exponentially as the major axis increases tending towards a specific minor axis value. It also falls away as the major and minor axis both increase. B) A bivariate histogram of the major/minor axis lengths of the negative FOV shown in Figure 2D. All clusters are far from the boundary of interest and there are very few of them.

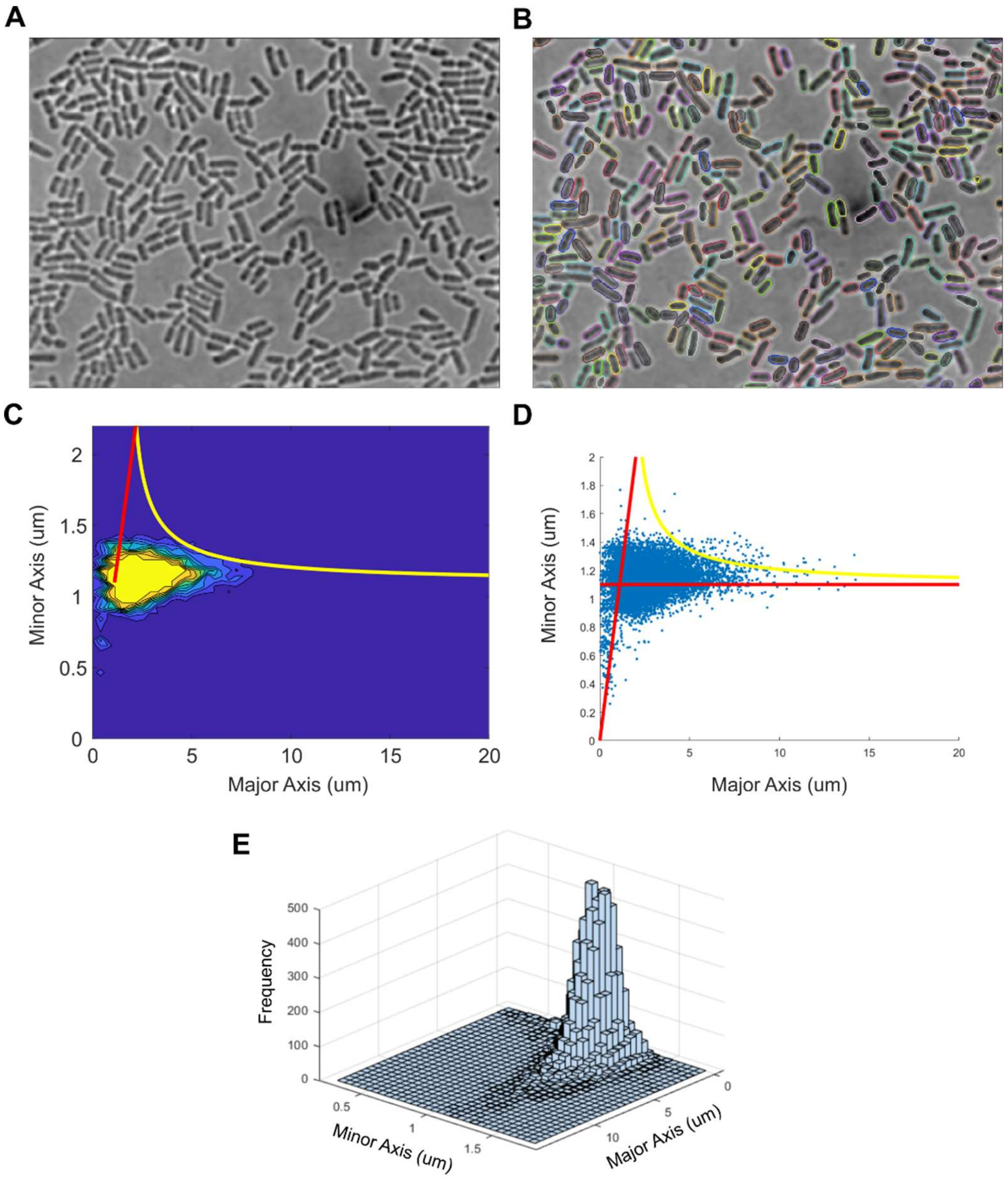

Sup. Fig. 3: Fake-phase imaging of E. coli cells fit against the predicted theory from pressure vessel analysis. A) Representative FOV of fake-phase imaging of E. coli cells. B) Segmentations of the FOV in A) from which the length and width of cells were calculated. C) A contour plot of the major and minor axis lengths showing the fit of the theoretical limit to experimentally measured cells with a maximum frequency of 100 for clarity. D) The major/minor axis scatter plot with lines at minor axis = 1.1 µm (red), major axis = minor axis (red) and with the line as given in equation 6 describing the derived allowable relation between major and minor axes with r_0 = 1.1 µm (yellow), showing the majority of points to be contained within the expected theoretical region. E) A bivariate histogram of the major and minor axis lengths showing a decrease in the width of the cells as their lengths increased.

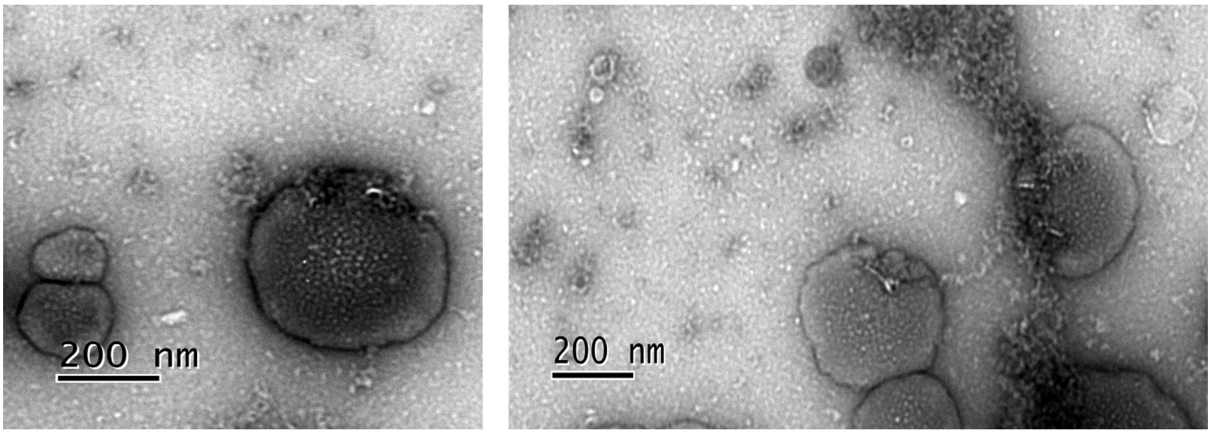

Sup. Fig. 4: Negative stain electron microscopy (EM) images provide a size estimate of RSV virions. Negative stain EM images of spherical RSV virions give a wide size distribution of 356 ± 130 nm, n=20. Scale bar 200 nm.
