# Supplementary material for: Engineering stress as a motivation for filamentous virus morphology
Source: Biophys Rep (N Y). 2024 Sep 10;4(4):100181. doi: 10.1016/j.bpr.2024.100181 (PMC11447354; doi:10.1016/j.bpr.2024.100181)
Supplement: Document S2. Article plus supporting material [file mmc2.pdf]

Biophysical Reports

REPORT

Engineering stress as a motivation for filamentous virus morphology

Andrew McMahon,[1,2,3,*] Swetha Vijayakrishnan,[4] Hafez El Sayyed,[1,2] Danielle Groves,[3] Michaela J. Conley,[4] Edward Hutchinson,[4] and Nicole C. Robb[1,3,*]

[1]Biological Physics Research Group, Clarendon Laboratory, Department of Physics, University of Oxford, Oxford, United Kingdom; [2]Kavli Institute for Nanoscience Discovery, Dorothy Crowfoot Hodgkin Building, University of Oxford, Oxford, United Kingdom; [3]Warwick Medical School, University of Warwick, Coventry, United Kingdom; and [4]MRC-University of Glasgow Centre for Virus Research, University of Glasgow, Glasgow, United Kingdom

ABSTRACT Many viruses are pleomorphic in shape and size, with pleomorphism often thought to correlate with infectivity, pathogenicity, or virus survival. For example, influenza and respiratory syncytial virus particles range in size from small spherical virions to filaments reaching many micrometers in length. We have used a pressure vessel model to investigate how the length and width of spherical and filamentous virions can vary for a given critical stress and fluorescence super-resolution microscopy along with image analysis tools to fit imaged influenza viruses to the model. We have shown that influenza virion dimensions fit within the theoretical limits of the model, suggesting that filament formation may be a way to increase an individual virus's volume without particle rupture. We have also used cryoelectron microscopy to investigate influenza and respiratory syncytial virus dimensions at the extrema of the model and used the pressure vessel model to explain the lack of alternative virus particle geometries. Our approach offers insight into the possible purpose of filamentous virus morphology and is applicable to a wide range of other biological entities, including bacteria and fungi.

WHY IT MATTERS Many viruses form particles that vary in size and shape, including small spheres or long filamentous structures that can reach many microns in length. While virion structure is of interest not only in the context of virus assembly but also because pleomorphic variations may correlate with infectivity and pathogenicity, filament formation is not well understood, and viral filaments are relatively understudied. In this paper, we used a pressure vessel model and microscopy to investigate the relationship between virion dimensions and engineering stress and concluded that viral filaments offer an optimal shape that allows viruses to have a larger volume than they could in any alternative geometry, thus offering new insight into viral filament formation.

INTRODUCTION

A pressure vessel is designed to hold liquids or gases at substantially higher or lower pressures than ambient pressure. A high pressure difference requires the correct design in order to avoid catastrophic failures, and pressure vessel models (1) are used to assess engineering stress development and whether vessels of a known geometry will be able to sustain the applied internal pressure. Stress is the force applied to a material divided by the area over which the force acts before deformation occurs. By equating the force due to pressure and the equal and opposite force from the stress, a relation between stress, pressure, and the dimensions of a given cross section of the container can be derived. In chemical engineering, pressure vessel models using theoretical reaction pressures and the critical stress of a material are used to determine the geometries of reaction vessels (size and wall thickness) so that their operation falls within allowed safety margins. We set out to investigate whether pressure vessel models could be applied to filamentous biological structures and provide insight into pressure-stress relations, using size data from images of viruses and bacteria to test our model.

A filamentous, or elongated, morphology is a common feature of many biological microorganisms and can be observed in multiple species of fungi, bacteria, and viruses. Viruses in particular can be extremely pleomorphic; in the case of influenza, particles can

Submitted April 5, 2024, and accepted for publication September 6, 2024.

*Correspondence: andrew.mcmahon@warwick.ac.uk or nicole.robb@warwick.ac.uk

Editor: Jorg Enderlein.

https://doi.org/10.1016/j.bpr.2024.100181

© 2024 The Author(s). Published by Elsevier Inc. on behalf of Biophysical Society.
This is an open access article under the CC BY license (http://creativecommons.org/licenses/by/4.0/).

range from small spheres in the region of 100 nm, to extended bacilliform particles, to long filaments, which reach tens of microns in length (2,3). The role of filaments in influenza is poorly understood, with current suggestions for why a filamentous morphology occurs including the increased ability of filaments to penetrate mucus layers (4); their increased directionality of travel on mucus layers compared to spherical forms, which enables filaments to infect cells further from the original host cell in a shorter time (5); their increased resilience to UV radiation (6); or their increased ability to evade antibodies due to their increased number of surface proteins per virion (7). In contrast, the larger surface area of a filament in comparison to a spherical particle means that significantly more proteins are required to form each progeny virion, and so overall, far fewer filamentous virions than spherical can be produced by each infected cell, which may explain why spherical virions are also formed during new virion production. In the case of respiratory syncytial virus (RSV), another pathogenic virus that has been observed to have a filamentous form, there is evidence that filamentous virions represent the infectious form of the virus required for fusion of the virus with the host cell (8).

In this study, we modeled filaments as cylindrical particles with hemispherical caps and used known results from thin-walled pressure vessels (1) to form a theory of how the major axis of a filament would change in relation to its minor axis given a critical stress (9). This derived relationship was tested using microscopy measurements of the length and width of influenza and RSV. We found that a pressure vessel model of a filament gave a quadratic relation between the major and minor axes that fitted well to the size measurements taken for both viruses, suggesting that filament formation may be a way of increasing the volume of a virus without leading to particle rupture. Overall, our modeling provides insights into how pressure and stress may influence the size and shape of viruses and other biological organisms.

MATERIALS AND METHODS

Calculating a theoretical major/minor axis relation

A thin wall pressure model was used to derive the three normal stresses i.e., stresses formed when the direction of the deforming force is perpendicular to the cross-sectional area of the body. The force due to pressure from the inside of a filament was equated with the force due to the stress in the walls of the filament due to Newton's third law, with the filament modeled as a cylinder with hemispherical caps. This gave principle stress/pressure relations for the longitudinal stress, σ_1, hoop stress, σ_2, and normal/external stress, σ_3:

$$\sigma_1 = \frac{P}{t}\frac{(\pi r^2 + 2Lr)}{(2\pi r + 2L)}, \tag{1}$$

$$\sigma_2 = P\frac{r}{2t}, \textit{and} \tag{2}$$

$$\sigma_3 = -P, \tag{3}$$

where P is the difference in pressure over the surface ($P_{internal} - P_{external}$), t is the thickness of the lipid layer, r is the radius of the cylinder and hemispheres, and L is the length of the cylinder. In the limit $L \rightarrow 0$, Eq. 1 tends to the normal hoop stress of a sphere, and, as $L \rightarrow \infty$, it tends to the hoop stress of a cylindrical pipe.

Assuming the breaking stress occurs when $\sigma_1 = \sigma_{max}$, $\sigma_2 = \sigma_{max}$, or $\sigma_3 = \sigma_{max}$ and using $r = r_\infty$ when $L \rightarrow \infty$, this gives us

$$r_\infty = \frac{\sigma_{max} t}{P}, \tag{4}$$

as σ_1 is always the greatest of the stress components.

This gives the relationship between L (the length of the cylindrical segment) and r, the radius, in terms of the radius of an infinitely long filament, r_∞, of

$$L = \frac{2\pi r r_\infty - \pi r^2}{2(r - r_\infty)}, \tag{5}$$

which, using the major axis length, $m_1 = L + 2r$, and the minor axis length, $m_2 = 2r = m_1 - L$, gives us the relation to compare experimentally between the major and minor axes:

$$m_1 = m_2 + \frac{\pi m_2 \left(r_\infty - \frac{{m_2}^2}{4} \right)}{m_2 - 2r_\infty} \tag{6}$$

Within the model, several assumptions were made.

1) The internal pressure was uniform across different virus particles of the same species.
2) The surface environment across the surface of virus particles was similar and the material was isotropic, and thus the maximum stress was constant.
3) The bending stress in the virus membrane was not considered in the thin wall approximation.
4) When cutting shapes to find a cross section, perpendicular lines on the neutral axis remain perpendicular after cutting.

Deriving a relation between spherical and filamentous virions with a non-negligible membrane thickness

Assuming the critical shear stress of the material is constant across filaments and spheres, we equated the maximum shear stress from Lamé's equations (10) for a sphere and a cylinder:

$$\frac{3p_o {b_{sphere}}^3 - p_i\left(2{a_{sphere}}^3 + {b_{sphere}}^3\right)}{2\left({a_{sphere}}^3 - {b_{sphere}}^3\right)} = \frac{p_i\left(a^2 + b^2\right) - 2p_o b^2}{b^2 - a^2}, \tag{7}$$

where b_{sphere} is the outer radius of spherical particles, a_{sphere} is the inner radius of spherical particles, b is the outer radius of filamentous particles, a is the inner radius of filamentous particles, p_o is the external particle pressure, and p_i is the internal particle pressure.

From this, we derived a relation between filament and spherical virion sizes that accounts for bending stresses given that the internal pressure is greater than the external pressure:

$$\frac{\left(b_{sphere}^3 + 2a_{sphere}^3\right)\left(b^2 - a^2\right)}{2\left(b_{sphere}^3 - a_{sphere}^3\right)\left(b^2 + a^2\right)} = 1 . \qquad (8)$$

RESULTS

The derivation of a relation between the major and minor axes of filaments

In order to investigate any physical motivation for filamentous morphologies, a pressure vessel model was formed. We did this by modeling the virus as a cylinder of length L and radius r with hemispherical caps of radius, r (Fig. 1). Taking a cross section of this and equating the force due to pressure with the oppositely directed force due to the stress in the walls of the filament, we could derive the known relation for a pressure vessel (materials and methods, Eq. 1). This pressure stress relation was valid for viruses of any size, from a sphere to a filament. The relation showed that the stress increased as the radius increased for spherical morphologies. However, for a filamentous morphology, the stress in the membrane did not continue to increase as the length increased. Instead, as the filament length tends to an infinite length, the stress tends to a value twice that of the spherical particle with the same radius (Fig. 1).

Once our pressure stress relation was formed, an experimentally testable relation was required. With the assumptions that there was a maximal allowed stress (as membranes rupture at high stress) and a constant internal pressure (as different pleiomorphic viruses bud from the same cells)m we derived a relation between the minor axis, $m_{2,}$ and the major axis, m_1, of viral particles (materials and methods, Eq. 6):

$$m_1 = m_2 + \frac{\pi m_2\left(r_\infty - \frac{{m_2}^2}{4}\right)}{m_2 - 2r_\infty}. \qquad (9)$$

This relation shows a quadratic relation between the major and minor axes for a given value of r_∞ and that as the major axis increases, the minor axis decreases. As m_1 tends to an infinite length, m_2 tends to half the value when $m_2 = m_1$. This provides us with a tractable relation that can be applied to the study of filamentous virus morphology.

The application of the pressure vessel model to biological images

To experimentally test the relation between major and minor axis lengths of filaments, we performed super-resolution imaging of the influenza virus A/Udorn/72, a strain with a well-characterized spherical and

Length	Shape	Pressure/stress relation
L=0	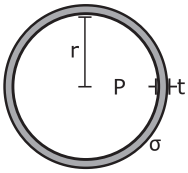	$P(\pi r^2) = \sigma(2\pi rt)$<br>$\sigma = P\frac{r}{2t}$
L>0	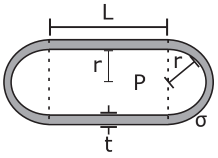	$P(\pi r^2+2rL) = \sigma(2\pi rt+2Lt)$<br>$\sigma = P\frac{\pi r^2+2rL}{2\pi rt+2Lt}$
L→∞	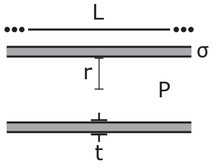	$P(2rL) = \sigma(2Lt)$<br>$\sigma = P\frac{r}{t}$

FIG. 1 Schematics of the cross sections of interest and the pressure/stress relations derived for increasing lengths of virus particles. Viruses are modeled as cylinders with hemispherical caps. For different lengths of cylinders, $L = 0$, $L > 0$, and $L \to \infty$, the pressure/stress relation is given along with the cross section of interest. In the limits of $L \to 0$ and $L \to \infty$, the model used for filamentous viruses tends to the known results for spheres and pipes of an infinite length.

filamentous phenotype (11). Virus particles were immobilized, fixed, and immunolabeled, and direct stochastic optical reconstruction microscopy (dSTORM) was carried out to obtain high-resolution reconstructed images, with a localization precision of 7.3 nm (Fig. 2 *A*) (11). Prior to this work, we carried out several control experiments, confirming that our methodology of drying and immunolabeling virus samples does not adversely influence the resulting images (11). Negative-stain electron microscopy images of our virus stocks also confirmed that virus particles were intact and did not appear to be aggregated (Fig. S1), giving us confidence that the majority of particles that we imaged were intact, non-aggregated virions. Both spherical (Fig. 2 *B*) and filamentous (Fig. 2 *C*) particles greater than 250 nm in length were observed in the resulting images. Minimal signal was observed in a negative control consisting of cell culture media lacking virus particles, confirming that the virus particles were specifically labeled (Fig. 2 *D*).

Measurements of the major and minor axes of particles were then taken from the super-resolution images by clustering the localizations using the DBScan algorithm and then fitting a confidence ellipse to the clustered data. A total of 41,754 viruses were measured from 46 fields of view (FOVs). The final bivariate histogram (Fig. 2 *E*) was produced by subtracting the negative FOV distributions (Fig. S2) from the positive FOV distributions. The bivariate histogram showed that the largest virions primarily appeared in two directions—along the line with the major axis equal to the minor axis and the line with the minor axis equal to 140 nm

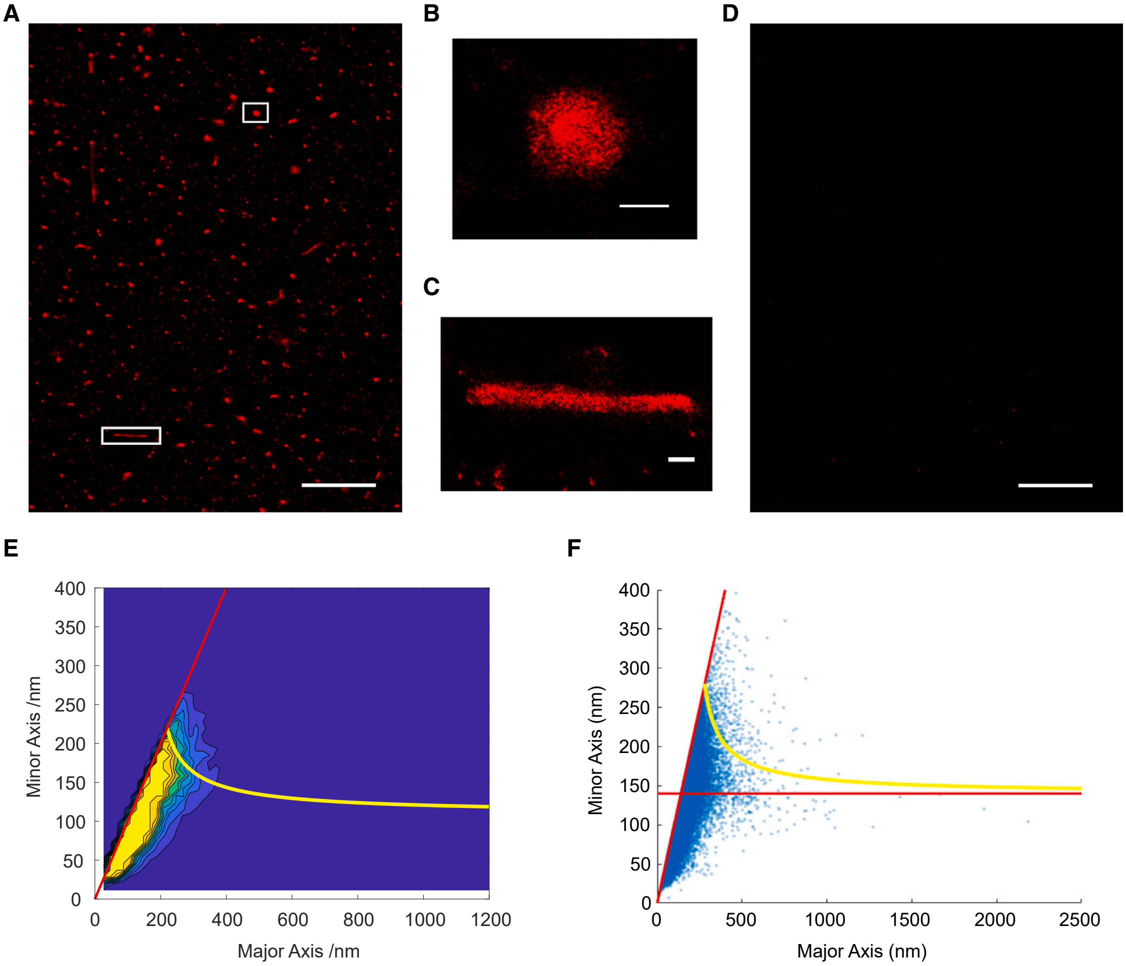

FIG. 2 Super-resolution imaging of spherical and filamentous influenza particles fit against the predicted theory from pressure vessel analysis. (*A*) A representative direct stochastic optical reconstruction microscopy (dSTORM) field of view (FOV) of labeled A/Udorn/72 influenza where the hemagglutinin protein is imaged in the red channel. Scale bar, 10 μm. (*B* and *C*) Zoomed-in images from (*A*) showing individual filaments and spherical particles. Scale bar, 200 nm. (*D*) A representative dSTORM FOV of a virus-negative sample, imaged in the red channel. Scale bar, 10 μm. (*E*) The major/minor axis contour plot with lines at major axis = minor axis (*red*) and with the line as given in Eq. 6 describing the derived allowable relation between major and minor axes with $r_0 = 70$ nm (*yellow*) with a maximum frequency of 100 for clarity. (*F*) The major/minor axis scatterplot with lines at minor axis = 140 nm (*red*) and major axis = minor axis (*red*) and with the line as given in Eq. 6 describing the derived allowable relation between major and minor axes with $r_0 = 70$ nm (*yellow*).

(Fig. 2 *E*). Additionally, a scatterplot of the major axis against the minor axis, with the lines $m_1 = m_2$ and $m_2 = 2r_\infty$ highlighted in red, and the derived relation between the minor axis and major axis (Eq. 6), with $r_\infty = 70$ nm plotted in yellow, showed the boundary of the points following the derived relation (Fig. 2 *F*).

To compare our findings on viruses to another biological system, we also imaged *E. coli* bacteria. *E. coli* is a straight, rod-shaped bacterium approximately 1–3 μm in length. In a natural, unstressed population, approximately 10% of the cells form filamentous structures (12); however, when they are stressed, the proportion of filamentous structures increases, which suggests that filaments may play a role in protecting the bacteria from predation, phagocytosis, and other stressors (12–14). Here, we imaged *E. coli* (Fig. S3 *A*) and segmented the individual bacteria within the images to measure their lengths and widths (Fig. S3 *B*). A bivariate histogram of these data showed a decrease in the width of the cells as their lengths increased, and a scatterplot with the modeled maximum size (*yellow*) with an r_0 of 1.1 μm (*red*) shows the main density of cells fitting the model with very good agreement (Fig. S3, *C*–*E*). Taken together, our results from images of both filamentous influenza virus and *E. coli* suggest that elongation of viruses and bacteria is accompanied by a narrowing of the particles along the minor axis, as predicted by our model.

Filamentous virus particles tend to half the width of spherical virions

To further investigate the application of our derived pressure stress model to filamentous viruses, we looked at the extrema of the model when the filaments were very long, i.e., microns in length (and the pressure stress relation can be modeled as that of a pipe), against which we could compare literature values for spherical virions. Our model predicted that the radius of filaments should be half the radius of the largest allowed spherical particles. In order to get experimental data to support our model, purified preparations of the A/Udorn/72 influenza virus and the A2 strain of RSV were imaged using cryoelectron tomography (cryo-ET) to produce tomograms of filaments with nanometer resolution (Fig. 3, *A* and *B*). By measuring the widths of individual filaments in the resulting images, we found the average external diameter (with and without surface glycoproteins), internal diameter, and thickness of the membrane for six influenza filaments and five RSV filaments.

This analysis provided measurements of 91.9 $\pm$ 1.1, 9.0 $\pm$ 0.6, 37.8 $\pm$ 0.4, and 55.8 $\pm$ 0.5 nm for the external diameter including glycoproteins, membrane thickness, internal diameter of the influenza filament membrane, and external diameter without glycoproteins, respectively (Fig. 3 *C*). This is in broad agreement with previously published values for the width of influenza filaments, for example, a study that measured 74.7 nm to the tips of the glycoproteins (15). Images of RSV filaments revealed that they were wider than influenza filaments, with average measurements for the external diameter including glycoproteins, membrane thickness, external diameter without glycoproteins, and internal diameter of 119.9 $\pm$ 1.5, 6.7 $\pm$ 0.8, 81.6 $\pm$ 1.2, and 94.9 $\pm$ 1.0 nm, respectively (Fig. 3 *D*). Similarly to the influenza measurements, these figures are in agreement with previously published values of between 71 and 166 nm for the width of RSV filaments (16).

Next, we compared our measured values from viral filaments with similar measurements for purified spherical influenza virions. Cryo-ET analysis of spherical A/WSN/33 influenza virions provided measurements of 110.6 $\pm$ 6.6, 7.6 $\pm$ 0.6, 67.5 $\pm$ 6.2, and 82.7 $\pm$ 5.9 nm for the external diameter including glycoproteins, membrane thickness, internal diameter of the membrane, and external diameter without glycoproteins, respectively ($n = 16$) (Fig. 3, *E* and *F*). We were unable to obtain cryo-ET images of purified RSV spheres because the purification procedure failed to produce intact spherical particles; however, negative-stain electron microscopy of unpurified RSV virions (Fig. S4) provided images of RSV particles with average diameters of 356 $\pm$ 130 nm ($n = 20$). Both sets of our measurements are in good agreement with previously published values for the diameter of spherical virions (84–170 nm for influenza (17) and 150–500 nm for RSV (18)). Comparison of our measured values for filaments with our values for spherical particle diameter provides good agreement with our model that predicts the filament membrane diameter to be approximately half the size of the maximum spherical membrane diameter. We also found that the membrane thickness of RSV was 7.6% of its diameter at its neutral axis (taken as the center of the viral membrane); for influenza, it was 19.6%. This puts RSV filaments comfortably within the thin wall approximation and influenza at the extreme of the model.

Although we have not included the effect of bending stress in our model, the measurements obtained from these cryo-ET images allow us to compare experimental and theoretical values for filaments and spheres taking bending stress into account. First, we used Lamé's equations to derive a relation between filament and spherical virion sizes that accounts for bending stresses for a constant critical shear stress, assuming that the internal pressure of virions is greater than the external pressure (see materials and methods). Using this relation and inputting the data from the electron tomograms of influenza (Fig. 3 *C* and *F*), we computed a value of 0.84 $\pm$ 0.08, which

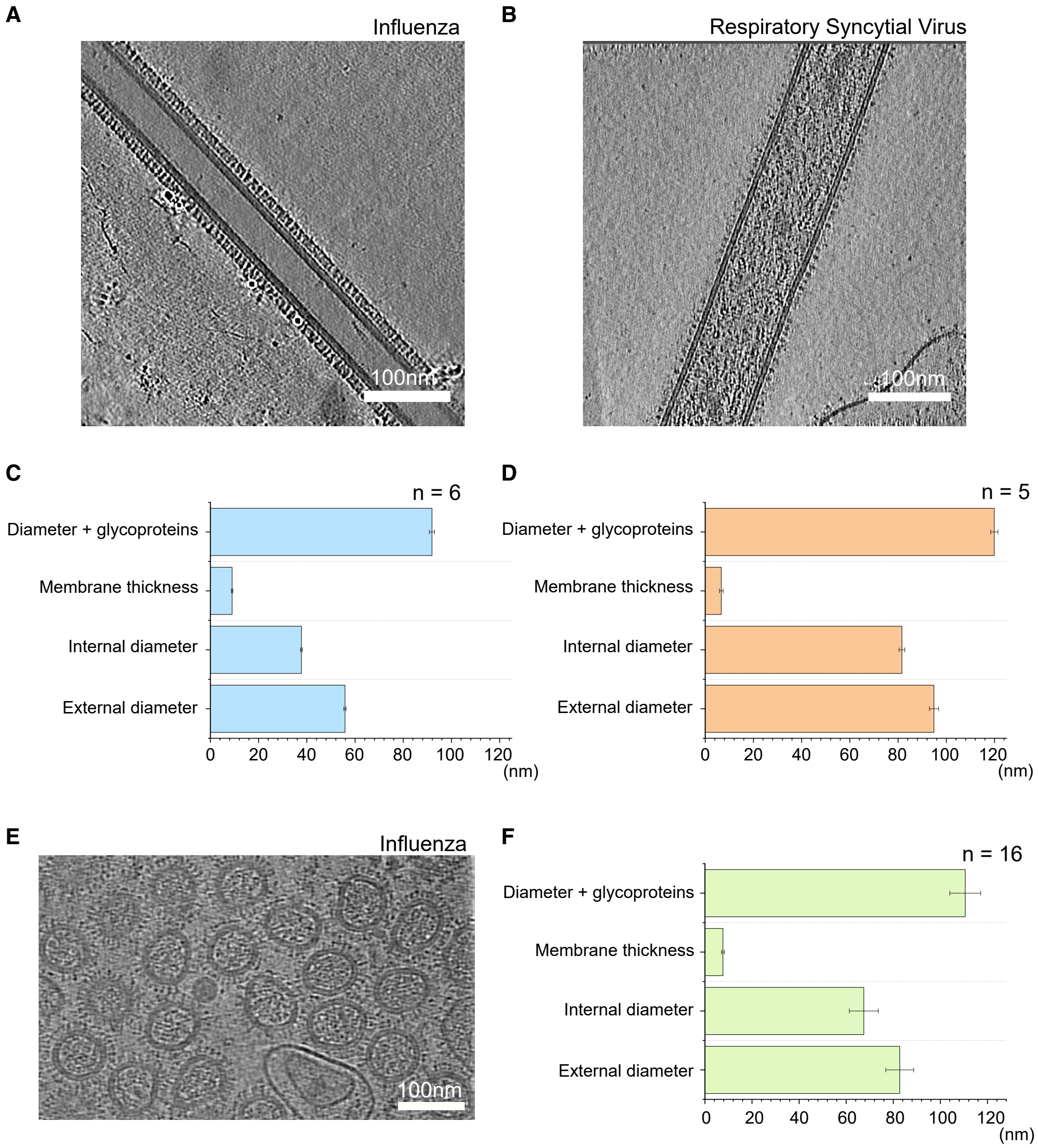

FIG. 3 Cryo-electron tomography of filamentous and spherical virus particles and measurements of their diameter and membrane thickness. (*A*) Representative tomogram of filamentous A/Udorn/72 influenza virus. Scale bar, 100 nm. (*B*) Representative tomogram of filamentous A2 respiratory syncytial virus. Scale bar, 100 nm. (*C* and *D*) Bar graphs of the average outer and inner diameters and the membrane thicknesses of the two filamentous viruses. (*E*) Representative tomogram of spherical A/WSN/33 influenza virus particles. Scale bar, 100 nm. (*F*) Bar graphs of the average outer and inner diameters and the membrane thicknesses of A/WSN/33 spheres.

is within two standard deviations of the expected value of one, suggesting that the dimensions of influenza virions fit the pressure vessel model well when taking bending stresses into consideration.

DISCUSSION

In this work, we have presented a pressure vessel model to explain the physical basis of filamentous virus morphology. A model was formed that related the stress, internal pressure, and length scales of a virus particle. It showed that the stress increased with the radius of a spherical virion and that a filament is a method of avoiding the size constraint that this imposes upon virion size due to the bursting stress of the membrane. We used microscopy images of influenza viruses to test our proposed model and confirmed that influenza virions tended to a decreased minor axis at large major axis length scales. Our

model's prediction that filamentous virions should have half the diameter of spherical virions also appears to fit experimental data for both influenza and a second filamentous virus, RSV. We were also able to provide similar data from images of the bacteria *E. coli*, which suggests that the model is applicable to other cells and biological entities. This may explain why some viral and bacterial species can grow up to tens of microns in length without rupture due to their internal pressure becoming too large.

Our derived model allows us to consider the potential different geometries that could be formed by virus particles. As smooth, symmetrical geometries minimize stress concentrations, we have considered the different geometries that could be formed as particles emerge symmetrically from the membrane in one, two, or three dimensions (1D, 2D, or 3D, respectively). In 1D, a filament is formed, in 2D a circular sheet, and in 3D a sphere (Fig. 4). We have excluded the possibility of nonuniform geometries, as these would produce local maxima in the stress of the membrane due to their local cross section and so are likely not formed due to the limitation of the rupture stress of the viral membrane. Our pressure model suggests that the increasing stress as spherical virus particles are enlarged in 3D to an infinite size would lead to rupture of the membrane, thus explaining the absence of large spherical viruses (Fig. 4, *top row*). Li et al. showed the lipid bilayer to be an easily deformable, nonrigid container (19), which suggests that 2D enlargement would also not be stable without further support to the membrane and that particles would "balloon" to a 3D spherical geometry upon budding from cells, also leading to rupture (Fig. 4, *middle row*). A filamentous structure, with elongation along just a single plane, will not encounter this problem (Fig. 4, *bottom row*). This model may therefore explain why virus particles exist as either small spheres or long filaments and have not been observed to bud in disk shapes along two principle directions.

Although our model predicts the allowed dimensions of the virus particles, it does not encompass the effects that the pool of host membrane at budding sites might have on these dimensions. This

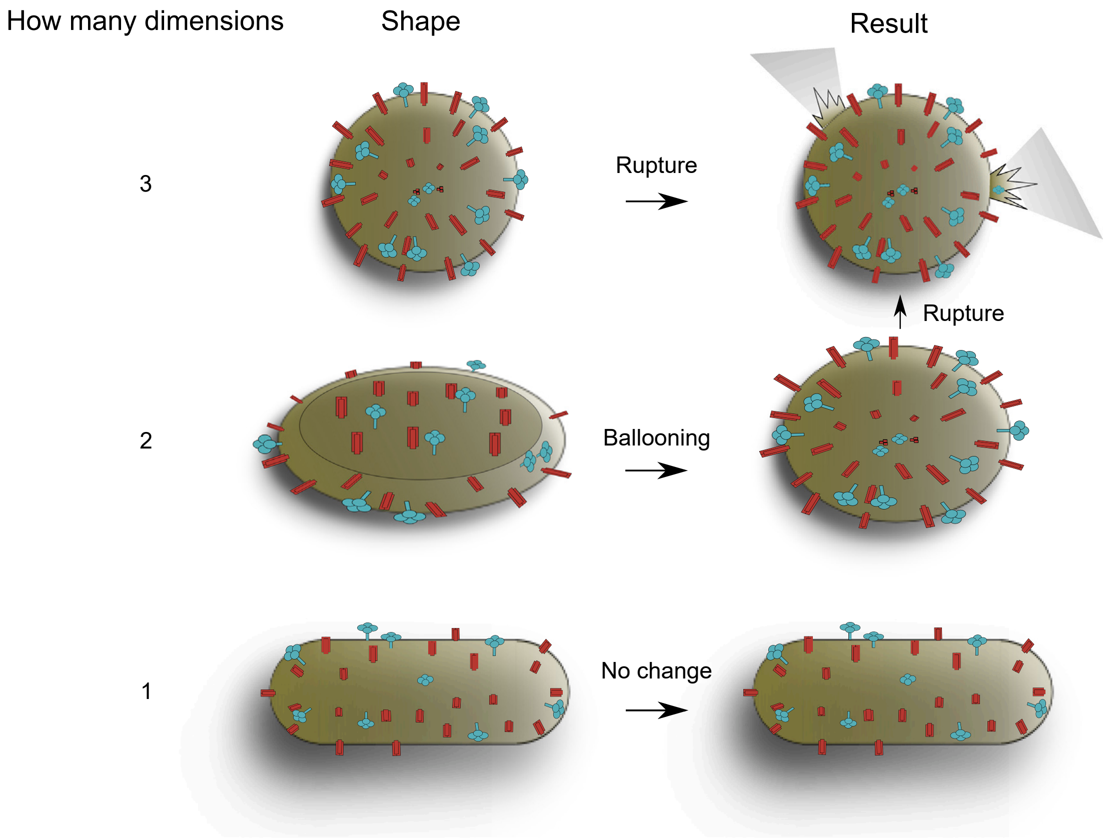

FIG. 4 Schematic of the proposed shapes formed from symmetric enlargement in three, two, or one dimensions and the outcome for virions with those geometries. The three possible shapes of the virus when they are enlarged symmetrically in either one, two, or three dimensions are shown. Three-dimensional enlargement is predicted to result in virion rupture when the maximum stress is reached, while growth in two dimensions is predicted to result in ballooning that will lead to a similar fate. Our data suggest that a filamentous shape with enlargement along a single plane is not likely to encounter this issue.

interaction may explain the ability of RSV viruses to form branched virions. Each individual branch in RSV has a very similar geometry to a viral filament, with the exception of the branching points, and so we believe that these are largely covered by our model. At the branch point, however, the geometry still appears to be relatively smooth (8), and so while these points are likely to have slightly higher stresses, we assume that the forces involved do not cause shear. The recently described "giant viruses," such as those belonging to the family *Mimiviridae*, which are distinguished by their large capsid diameters (200–400 nm), could potentially pose an exception to our model; however, we note some points of potential importance, namely that giant viruses are contained within rigid protein capsids and that exit from the host cell occurs via lysis rather than budding.

Our model has a number of limitations, including the following: the pressure throughout filaments and particles may not be fully uniform; structural proteins within the lipid membrane may change the maximum stress of filaments (assumed to be a constant); the walls have a finite thickness, and so the assumption that the radius of the lipid layer is equal to the radius of the inside of the virus is not perfect; bending stress is not included in the model (see below); and the model cannot explain the mechanism of filament formation or why some strains of viruses form filaments but others do not. In spite of these caveats, our model suggests filament formation to be a method through which enveloped viruses can become larger than the limit placed upon them from the stress of a lipid bilayer. As larger viruses require more material from the cell to assemble, and thus their formation decreases the total number of virions produced, this suggests that filament formation must offer the virus an advantage. Several studies have suggested that this is indeed the case, with filamentous viruses having an advantage in transmission (e.g., through containing multiple copies of the genome) (6) by increasing the virus particle surface area and thus making filaments less susceptible to neutralizing antibodies (7), increasing the ability of filaments to penetrate mucus layers (4), or increasing the resilience to UV radiation (6).

As mentioned above, the full complexity of the viral system is not completely captured by our model and images. Prior to rupture, the membrane of the virus is expected to detach from the underlying protein matrix layer. We would expect that the loss of the matrix layer will result in a decrease in the critical shear stress for the virion, which in turn would increase the probability of rupture. We have also not included bending stress in our model, which would be expected to result in a higher stress in particles with a greater curvature than predicted in our calculations, which would in turn lead to a smaller diameter of filamentous virions. In order to investigate this further, we derived a relation between the dimensions of spherical and filamentous virions taking bending stress into consideration and compared the theoretical values obtained to our experimental cryo-ET measurements of influenza virions. The small difference between theoretical and experimental values suggests that the dimensions of influenza virions do fit the pressure vessel model well when taking bending stresses into consideration. Due to the relatively low-throughput nature of the cryo-ET method, we have only carried out these measurements on a relatively small number of virus particles and may, therefore, have not captured the full variability of these viruses; however, we are confident that the inclusion of further images will not alter our main conclusion: that engineering stress is a key motivation for filamentous virus morphology.

In conclusion, modeling of viruses as a thin-walled pressure vessel with a maximum stress leads to the conclusion that filaments are formed as a method of increasing the size of virions while staying below this maximum stress. The model can be used to explain why virus particles exist as either small spheres or long filaments rather than in nonuniform geometries or disk-shaped particles. Furthermore, the model is broadly applicable to a wide range of biological organisms that form elongated filamentous structures.

ACKNOWLEDGMENTS

This research was supported by Royal Society Dorothy Hodgkin Research Fellowship DKR00620 and Research Grant for Research Fellows RGF\R1\180054 (to N.C.R.), Medical Research Council core grant MC_UU_12014/7 (to S.V.), UK Biotechnology and Biological Sciences Research Council grant BB/X015637/1 (to H.E.S.), a Medical Research Council-Doctoral Training Partnership-funded studentship (to D.G.), and a Medical Research Council Career Development Award MR/N008618/1 (to E.H.). We would like to thank Dr. James Streetly and Prof. David Bhella for assistance with cryo-tomography and Dr. Saskia Bakker for assistance with the negative-stain electron microscopy.

AUTHOR CONTRIBUTIONS

A.M. conceived the project, acquired data, performed the analysis, and drafted the manuscript; H.E.S. contributed to image acquisition; S.V., M.J.C., and D.G. acquired data and contributed to image acquisition; E.H. contributed to funding acquisition and supervision; and N.C.R. contributed to funding acquisition and supervision, drafted figures and revised the manuscript.

DECLARATION OF INTERESTS

The authors declare no competing interests.

SUPPORTING MATERIAL

Supplemental information can be found online at https://doi.org/10.1016/j.bpr.2024.100181.

REFERENCES

1. Philpot, T. A. 2011. Mechanics of materials : an integrated learning system, 2nd ed. Wiley. xvii, Hoboken, NJ, p. 767.
2. Mosley, V. M., and R. W. Wyckoff. 1946. Electron micrography of the virus of influenza. *Nature.* 157:263.
3. Laine, R. F., G. Goodfellow, ..., C. F. Kaminski. 2018. Structured illumination microscopy combined with machine learning enables the high throughput analysis and classification of virus structure. *Elife.* 7:e40183.
4. Roberts, P. C., and R. W. Compans. 1998. Host cell dependence of viral morphology. *Proc. Natl. Acad. Sci. USA.* 95:5746–5751.
5. Vahey, M. D., and D. A. Fletcher. 2019. Influenza A virus surface proteins are organized to help penetrate host mucus. *Elife.* 8:e43764.
6. Smirnov Yu, A., M. A. Kuznetsova, and N. V. Kaverin. 1991. The genetic aspects of influenza virus filamentous particle formation. *Arch. Virol.* 118:279–284.
7. Li, T., Z. Li, T. Ivanovic..., 2021. The shape of pleomorphic virions determines resistance to cell-entry pressure. *Nature Microbiology.* 6:617–629.
8. Ke, Z., R. S. Dillard, ..., E. R. Wright. 2018. The Morphology and Assembly of Respiratory Syncytial Virus Revealed by Cryo-Electron Tomography. *Viruses.* 10:446.
9. Megson, T. H. G. 1996. Structural and stress analysis. Arnold, London, Halstead, p. 641.
10. MyDatabook. 2024. Stress for Thick Walled Cylinders using Lamé's Equations. Available from. https://www.mydatabook.org/solid-mechanics/stress-for-thick-walled-cylinders-and-spheres-using-lames-equations/#google_vignette.
11. McMahon, A., R. Andrews, ..., N. C. Robb. 2023. High-throughput super-resolution analysis of influenza virus pleomorphism reveals insights into viral spatial organization. *PLoS Pathog.* 19:e1011484.
12. Cushnie, T. P. T., N. H. O'Driscoll, and A. J. Lamb. 2016. Morphological and ultrastructural changes in bacterial cells as an indicator of antibacterial mechanism of action. *Cell. Mol. Life Sci.* 73:4471–4492.
13. Jaimes-Lizcano, Y. A., D. D. Hunn, and K. D. Papadopoulos. 2014. Filamentous Escherichia coli cells swimming in tapered microcapillaries. *Res. Microbiol.* 165:166–174.
14. Hahn, M. W., and M. Höfle. 1998. Grazing Pressure by a Bacterivorous Flagellate Reverses the Relative Abundance of Comamonas acidovorans PX54 and Vibrio Strain CB5 in Chemostat Cocultures. *Appl. Environ. Microbiol.* 64:1910–1918.
15. Vijayakrishnan, S., C. Loney, ..., D. Bhella. 2013. Cryotomography of budding influenza A virus reveals filaments with diverse morphologies that mostly do not bear a genome at their distal end. *PLoS Pathog.* 9:e1003413.
16. Kiss, G., J. M. Holl, ..., E. R. Wright. 2014. Structural analysis of respiratory syncytial virus reveals the position of M2-1 between the matrix protein and the ribonucleoprotein complex. *J. Virol.* 88:7602–7617.
17. Harris, A., G. Cardone, ..., A. C. Steven. 2006. Influenza virus pleiomorphy characterized by cryoelectron tomography. *Proc. Natl. Acad. Sci. USA.* 103:19123–19127.
18. Bachi, T., and C. Howe. 1973. Morphogenesis and ultrastructure of respiratory syncytial virus. *J. Virol.* 12:1173–1180.
19. Li, S., F. Eghiaian, ..., I. A. T. Schaap. 2011. Bending and puncturing the influenza lipid envelope. *Biophys. J.* 100:637–645.

Biophysical Reports, Volume *4*

Supplemental information

Engineering stress as a motivation for filamentous virus morphology

Andrew McMahon, Swetha Vijayakrishnan, Hafez El Sayyed, Danielle Groves, Michaela J. Conley, Edward Hutchinson, and Nicole C. Robb

Supplementary Material

Engineering stress as a motivation for filamentous virus morphology

Andrew McMahon[1,2,3,*], Swetha Vijayakrishnan[4], Hafez El Sayyed[1,2], Danielle Groves[3], Michaela J. Conley[4], Edward Hutchinson[4], and Nicole C. Robb[1,3,*]

[1]Biological Physics Research Group, Clarendon Laboratory, Department of Physics, University of Oxford, Oxford, OX1 3PU, United Kingdom

[2]Kavli Institute for Nanoscience Discovery, Dorothy Crowfoot Hodgkin Building, University of Oxford, South Parks Rd, Oxford, OX1 3QU, United Kingdom

[3]Warwick Medical School, University of Warwick, Coventry, CV4 7AL, United Kingdom

[4]MRC-University of Glasgow Centre for Virus Research, University of Glasgow, Glasgow, G61 1QH, United Kingdom

*To whom correspondence should be addressed: Nicole.Robb@warwick.ac.uk and Andrew.McMahon@warwick.ac.uk

Supplementary Methods

Viruses. The influenza strain A/Udorn/72 (H3N2) (Udorn) was a kind gift from Professor Ervin Fodor, University of Oxford and was grown in Madin-Darby canine kidney (MDCK) cells as previously described [1]. Udorn virions have been shown to exhibit both a filamentous and spherical morphology [1]. Respiratory Syncytial Virus strain A2 (RSV) (ATCC, VR-1540) was grown in HEp-2 cells as previously described [2]. RSV virions have also been shown to exhibit both a filamentous and spherical morphology [3]. Spherical influenza A/WSN/33 (H1N1) (WSN) was prepared by reverse genetics, as previously described [4], and propagated on MDCK cells.

Super-resolution imaging and analysis. A/Udorn/72 influenza virus particles within cell supernatant (or cell media only as a negative control) were imaged using direct stochastic optical reconstruction microsopy (dSTORM) and analysed as described previously [1]. Briefly, viruses were dried onto a poly-L-lysine treated glass coverslip, fixed with 4% formaldehyde (Thermo Scientific), permeabilized with 0.5% Triton-X-100 (MP Biomedicals) and immunolabelled. We previously found that including a permeabilization step resulted in a higher number of labelled particles in our images, regardless of whether the target protein was internal or external [1]. A primary antibody against the haemagglutinin protein (Hc83x, a kind gift from Stephen Wharton, Francis Crick Institute) and a secondary antibody labelled with Alexa647 (Invitrogen) were used. The viruses were imaged on a commercially available Nanoimager fluorescence microscope (Oxford Nanoimaging) using total internal reflection fluorescence (TIRF) microscopy. The laser illumination was focused at an angle of 53° with respect to the default position. Images of a field of view (FOV) measuring 80 x 50 µm were taken with an exposure time of 30 ms at a laser intensity of up to 780 kW/cm^2, and movies of between 5,000 and 15,000 frames were taken.

The signals in each FOV were detected and fit to a 2D Gaussian in each frame using the analysis software from Oxford Nanoimaging, drift corrected via phase correlation between frames, and exported. Each FOV was clustered using the sklearn clustering library implementation of the DBScan clustering algorithm with an epsilon of 30 nm and a minimum cluster size of 200 nm [1]. Using the confidence_ellipse library, a confidence ellipse was fit with the standard deviation of the ellipse set as 2.0. For the width of particles, the scipy.spatial implementation of the convex hull method was used. The major and minor axes of the ellipse were taken as the size of the viral particles.

Cryo-electron tomograhy imaging and analysis. Preparation of grids for cryo-electron tomography (cryo-ET) and imaging was carried out as described previously [5]. Briefly, Madin-Darby canine kidney (MDCK) and adenocarcinomic human alveolar epithelial (A549) cells were directly seeded on cryo-EM grids prior to infection with human RSV (strain A2-RSV) or A/Udorn/72 (H3N2) at an MOI of 1 and incubated for a further 24 h (Udorn) or 72 h (RSV A2). Stocks of A2-RSV and Udorn virus were produced in house as previously described [5, 6]. Stocks of WSN were prepared for cryo-electron tomography as previously described [7]. Briefly, twenty confluent T150 flasks of MDCK cells were infected with WSN at an MOI of 0.001 PFU/cell and maintained in serum-free DMEM at 37°C. After two days, the growth medium was clarified twice by low-speed centrifugation and then virions were concentrated by ultracentrifugation at 112,000 × *g* for 90 minutes at 4°C through a cushion of 10% iodixanol (supplied as OptiPrep, Sigma) in NTC (0.1 M NaCl, 20 mM Tris-HCl pH 7.4, 5 mM $CaCl_2$). The pellet was resuspended in NTC and separated through a 10 – 35% gradient of iodixanol in NTC, prepared using a Gradient Master (BioComp Instruments), by ultracentrifugation at 4 °C for 150 min at 209,000 × *g*. WSN virions, visible as a thick milky band on the gradient, were drawn off by side-puncture, pelleted by ultracentrifugation through NTC at 4 °C for 60 min at 154,000 × *g*, and resuspended in 80 µl NTC.

Grids were plunge frozen as follows. Briefly, RSV-infected and Udorn-infected grids were supplemented with 3 µl of 5 nm colloidal gold bead suspension, and purified WSN virus (4 µl) was

supplemented with 1 µl of 10 nm colloidal gold bead suspension (BBI Solutions, United Kingdom). Grids were then transferred to a Vitrobot Mk IV (Thermo Fisher Scientific), blotted for four or five seconds and immediately plunged into a bath of liquid ethane. Tilt-series imaging was performed at the UK electron bio-imaging centre at Diamond Light Source (eBIC) on a Titan Krios microscope (Thermo Fisher Scientific, Germany) equipped with a Gatan BioQuantum K2 energy filtered direct detection camera, and at the Scottish Centre for Macromolecular imaging (SCMI) on a JEOL CRYO ARM 300 (JEOL, Japan) equipped with an energy filter and a DE64 direct electron camera (Direct Electron, USA). Tilt series alignment and tomogram reconstruction was performed and visualized using the IMOD software package [8]. Reconstruction was carried out using weighted back projection followed by denoising using Topaz [9]. Figures were prepared by averaging 10 tomogram sections using IMOD's 3dmod slicer routine.

The contrast of each tomogram taken was adjusted to make the boundaries of the filaments clear. The outer and inner diameters of each filament in the acquired tomograms were measured in 5 different locations with the line tool of ImageJ. These were used to calculate the average width of each filament and the error in these measurements. From the diameter information, the wall thickness was calculated with the error in wall thickness calculated through error propagation.

Negative Stain Electron Microscopy. Carbon coated formvar grids were floated on droplets of the virus samples for 5 min followed sequentially by 5 min on a droplet of water and then on 1% uranyl acetate. The grids were blotted and allowed to dry before examination in a JEOL 2100 Plus microscope operating at 200 kV. We measured the size (average of major and minor axes) of particles using the wand tool in ImageJ.

Bacteria imaging and analysis. Images of Escherichia coli (E. coli) bacteria were acquired as described previously [10]. Briefly, bacteria were grown to their exponential phase when the OD600 was measured to be ~0.2 and imaged using brightfield microscopy. Brightfield images were illuminated using a white LED light source (CoolLED pE-100). Collected light was passed back through the multi-notch filter with transmission windows at 439±15 nm, 21±17 nm, and 605±25 nm, with an achromatic doublet lens (AC508-300-A, ThorLabs, Newton, New Jersey, USA) forming an image on an EMCCD camera (iXon 897 Ultra, Andor Technology Ltd, Belfast, UK). Image acquisition was performed using the software package Andor SOLIS (Andor Technology, Belfast, UK).

Bacterial image analysis was carried out using bacterial segmentation software. Cells were segmented using a convolutional neural network trained on manually labelled data, dividing and incomplete cells were excluded from the segmentation, and then imperfect segmentations were cleaned up manually. From the segmentation mask, the lengths and widths of cells were extracted.

Supporting References

1. McMahon, A., et al., *High-throughput super-resolution analysis of influenza virus pleomorphism reveals insights into viral spatial organization.* PLoS Pathog, 2023. **19**(6): p. e1011484.
2. Haney, J., et al., *Coinfection by influenza A virus and respiratory syncytial virus produces hybrid virus particles.* Nat Microbiol, 2022. **7**(11): p. 1879-1890.
3. Ke, Z., et al., *The Morphology and Assembly of Respiratory Syncytial Virus Revealed by Cryo-Electron Tomography.* Viruses, 2018. **10**(8).
4. Fodor, E., et al., *Rescue of influenza A virus from recombinant DNA.* J Virol, 1999. **73**(11): p. 9679-82.

5. Vijayakrishnan, S., et al., *Cryotomography of budding influenza A virus reveals filaments with diverse morphologies that mostly do not bear a genome at their distal end.* PLoS Pathog, 2013. **9**(6): p. e1003413.
6. Conley, M.J., et al., *Helical ordering of envelope-associated proteins and glycoproteins in respiratory syncytial virus.* EMBO J, 2022. **41**(3): p. e109728.
7. Hutchinson, E.C. and M. Stegmann, *Purification and Proteomics of Influenza Virions.* Methods Mol Biol, 2018. **1836**: p. 89-120.
8. Kremer, J.R., D.N. Mastronarde, and J.R. McIntosh, *Computer visualization of three-dimensional image data using IMOD.* J Struct Biol, 1996. **116**(1): p. 71-6.
9. Bepler, T., et al., *Topaz-Denoise: general deep denoising models for cryoEM and cryoET.* Nat Commun, 2020. **11**(1): p. 5208.
10. El Sayyed, H., et al., *Single-molecule tracking reveals the functional allocation, in vivo interactions, and spatial organization of universal transcription factor NusG.* Mol Cell, 2024. **84**(5): p. 926-937 e4.

Supplementary Figures

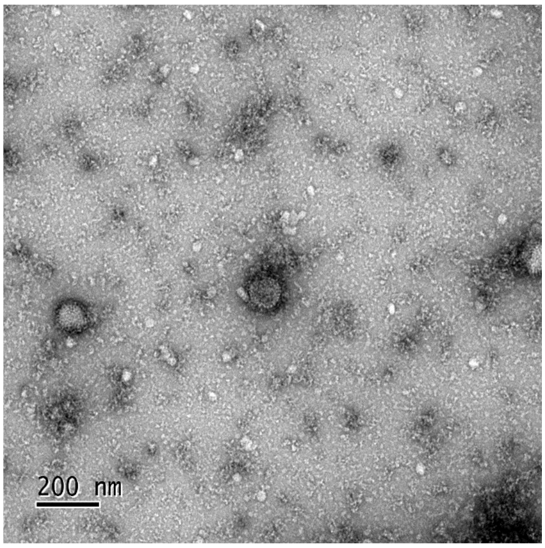

Sup. Fig. 1: Negative stain electron microscopy (EM) image of virus particles showing them to be intact and not aggregated. Negative stain EM image of A/Udorn/72 virions. Scale bar 200 nm.

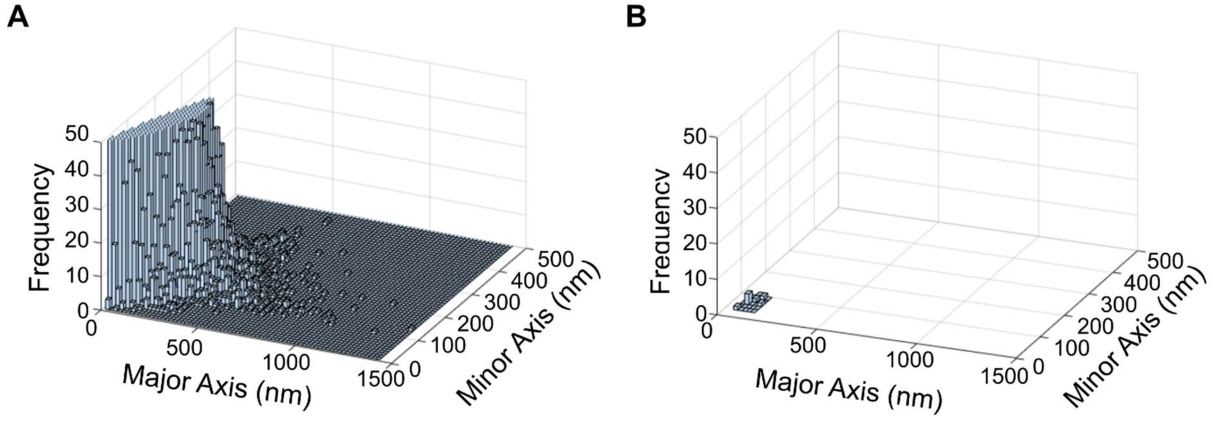

Sup. Fig. 2: Super-resolution imaging of spherical and filamentous influenza particles fit against the predicted theory from pressure vessel analysis. A) A bivariate histogram of the major/minor axis lengths. The frequency decreases exponentially as the major axis increases tending towards a specific minor axis value. It also falls away as the major and minor axis both increase. B) A bivariate histogram of the major/minor axis lengths of the negative FOV shown in Figure 2D. All clusters are far from the boundary of interest and there are very few of them.

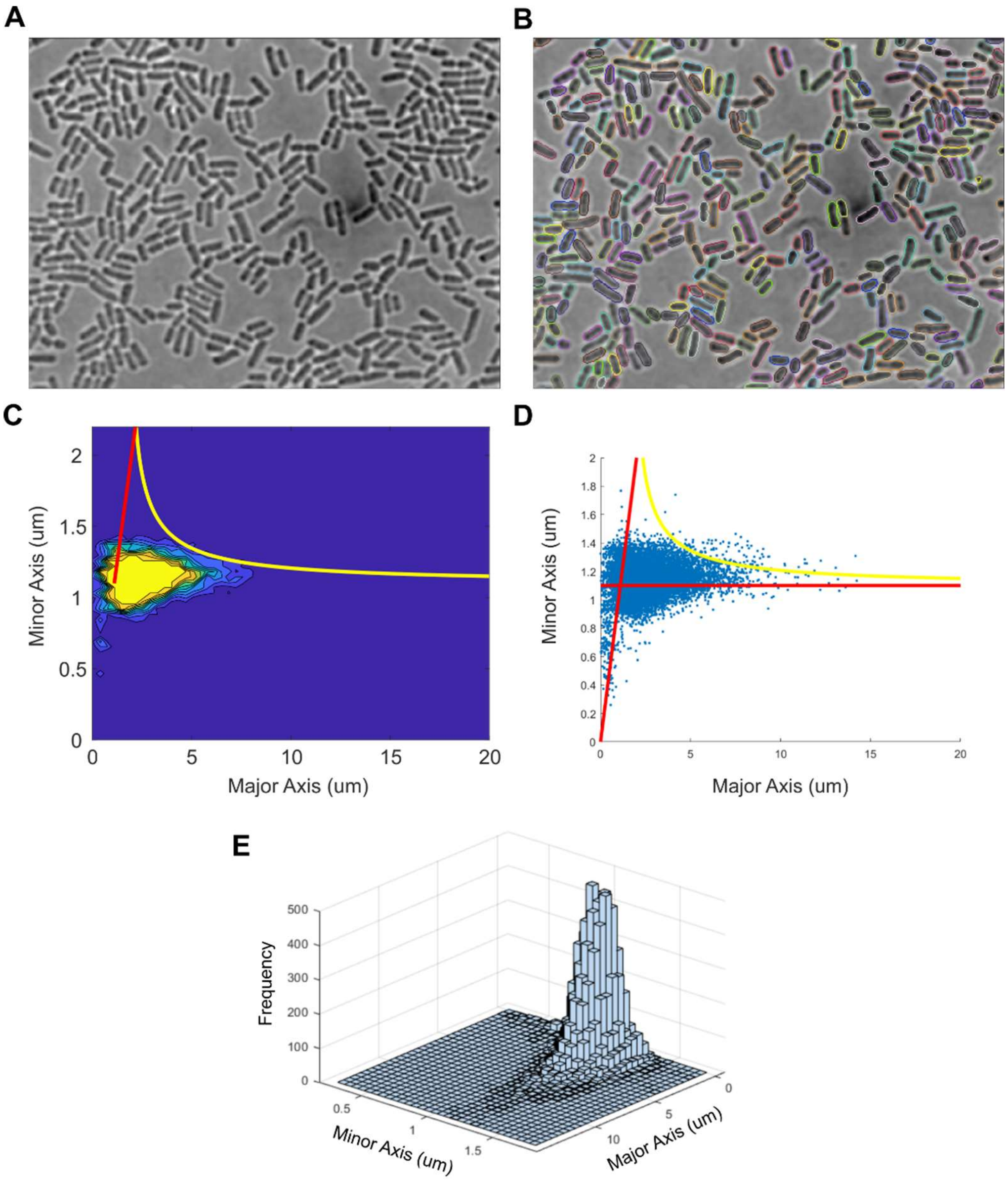

Sup. Fig. 3: Fake-phase imaging of E. coli cells fit against the predicted theory from pressure vessel analysis. A) Representative FOV of fake-phase imaging of E. coli cells. B) Segmentations of the FOV in A) from which the length and width of cells were calculated. C) A contour plot of the major and minor axis lengths showing the fit of the theoretical limit to experimentally measured cells with a maximum frequency of 100 for clarity. D) The major/minor axis scatter plot with lines at minor axis = 1.1 µm (red), major axis = minor axis (red) and with the line as given in equation 6 describing the derived allowable relation between major and minor axes with r_0 = 1.1 µm (yellow), showing the majority of points to be contained within the expected theoretical region. E) A bivariate histogram of the major and minor axis lengths showing a decrease in the width of the cells as their lengths increased.

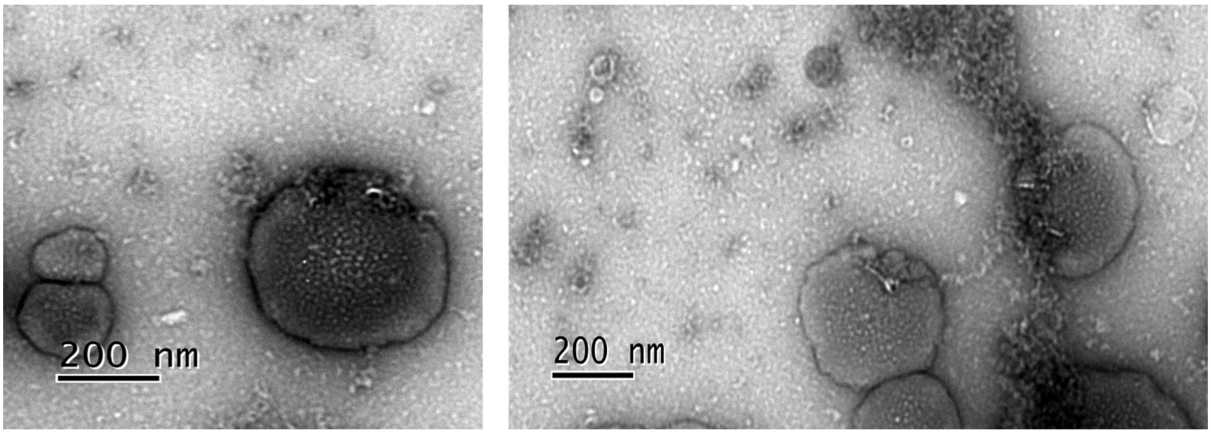

Sup. Fig. 4: Negative stain electron microscopy (EM) images provide a size estimate of RSV virions. Negative stain EM images of spherical RSV virions give a wide size distribution of 356 ± 130 nm, n=20. Scale bar 200 nm.
